# Red LED Light Acts on the Mitochondrial Electron Chain of Mammalian Sperm via Light-Time Exposure-Dependent Mechanisms

**DOI:** 10.3390/cells9122546

**Published:** 2020-11-26

**Authors:** Olga Blanco-Prieto, Jaime Catalán, Lina Trujillo-Rojas, Alejandro Peña, Maria Montserrat Rivera del Álamo, Marc Llavanera, Sergi Bonet, Josep Maria Fernández-Novell, Marc Yeste, Joan E. Rodríguez-Gil

**Affiliations:** 1 Unit of Animal Reproduction, Department of Animal Medicine and Surgery, Faculty of Veterinary Medicine, Autonomous University of Barcelona, Bellaterra, E-08193 Cerdanyola del Vallès, Spain; obprieto@gmail.com (O.B.-P.); dr.jcatalan@gmail.com (J.C.); linatrujillor@gmail.com (L.T.-R.); alex.pena@uab.cat (A.P.); mariamontserrat.rivera@uab.cat (M.M.R.d.Á.); 2 Biotechnology of Animal and Human Reproduction (TechnoSperm), Institute of Food and Agricultural Technology, University of Girona, E-17003 Girona, Spain; marc.llavanera@udg.edu (M.L.); sergi.bonet@udg.edu (S.B.); 3 Unit of Cell Biology, Department of Biology, Faculty of Sciences, University of Girona, E-17003 Girona, Spain; 4 Department of Biochemistry and Molecular Biology, Faculty of Biology, University of Barcelona, E-08028 Barcelona, Spain; jmfernandeznovell@ub.edu

**Keywords:** sperm, photobiology, mitochondrial function, mammalian, oligomycin A, FCCP

## Abstract

This work analyzes the effects of red LED light on mammalian sperm mitochondrial function, using the pig as an animal model. Liquid-stored pig semen was stimulated with red-light for 1, 5 and 10 min in the presence or absence of oligomycin A, a specific inhibitor of mitochondrial ATP synthase, or carbonyl cyanide 4-(trifluoromethoxy)phenylhydrazone (FCCP), a specific disruptor of mitochondrial electron chain. Whereas exposure for 1 and 5 min significantly (*p* < 0.05) decreased total motility and intracellular ATP levels, irradiation for 10 min induced the opposite effect. Oligomycin A abolished the light-effects on intracellular ATP levels, O_2_ consumption and mitochondrial membrane potential, whereas compared to non-irradiated samples, FCCP significantly (*p* < 0.05) increased O_2_ consumption when sperm were irradiated for 1 min. Both oligomycin A and FCCP significantly (*p* < 0.05) decreased total motility. Red-light increased cytochrome *c* oxidase activity with a maximal effect after 5 min of irradiation, which was abolished by both oligomycin A and FCCP. In conclusion, red-light modulates sperm mitochondrial function via electron chain activity in an exposition, time-dependent manner.

## 1. Introduction

Since about 95% of swine farms in Western countries use Artificial Insemination (AI) with liquid-stored pig semen (resulting in an estimated mean fertility of 90%), its optimization in that sector is crucial [1,2,3]. As any technique aimed at increasing reproductive performance needs to be contemplated, several approaches have been undertaken over recent years. Among these approaches is red-light irradiation, which increases both farrowing rates and litter sizes showing, however, significant variations across farms worldwide [4,5]. In addition, the literature remains inconsistent on how light-irradiation affects sperm function and its ability to modulate in vitro capacitation [4,6,7]. In this context, additional research about the mechanism/s underlying the mammalian sperm response to red-light is warranted.

At present, three potential mechanisms have been suggested to be related to the response of mammalian sperm to red-light (reviewed in [8]). The first one hypothesizes that light interacts with plasma membrane receptors, such as those belonging to the Transient Receptor Potential (TRP) family, linked to thermotaxis. The TRP family is a highly heterogeneous group and only proteins included in vanilloid TRP (TRPV), ankyrin TRP (TRPA) and melastanin TRP (TRPM) subfamilies are involved in the control of thermotaxis [9]. As part of the first subfamily, TRPV4 has been demonstrated to be the most important temperature-sensitive ion channel in mammalian sperm [10]. The second mechanism is related to the ability of light to interact with specific receptors belonging to the opsins family. In this respect, not only has rhodopsin, a retinal-dependent opsin sensitive to red-light [11], been identified in mouse and human spermatozoa [12], but also other opsins, such as blue light-sensitive melanopsin, ultraviolet-responsive neuropsin and PNN3/encephalopsin [12,13,14,15,16,17,18,19,20]. While the exact function of these opsins in mammalian sperm still remains unknown, mounting evidence suggests that these proteins—especially rhodopsin—are involved, together with TRP proteins, in the modulation of sperm thermotaxis [12,20]. Finally, the third mechanism contemplates the direct effect of irradiation on intracellular light-sensitive proteins (photosensitizers), such as mitochondria cytochromes, which are crucial for the regulation of the overall mitochondrial function. Cytochromes are essential components of the mitochondrial electron chain and play a vital role in the control of oxidative phosphorylation, generation of reactive oxygen (ROS) and apoptosis [21]. A common characteristic of cytochromes is the presence of a heme group, which accepts and donates electrons across the electron chain [22,23]. Since the heme group is light-sensitive, cytochromes are known to react against specific light wavelengths depending on their structure [24]. Thus, whereas cytochrome P450 has a higher absorption peak at 450 nm, other cytochrome complexes, such as cytochrome *c*, has two peaks (at 610–630 nm and 660–680 nm) [24].

Against this background, this study aimed to examine how irradiation of mammalian sperm with red-light affects mitochondrial function, since this is one of the hypothesized mechanisms of action. With this purpose, and using the pig as a model, semen stored at 17 °C was irradiated with red-light for 1, 5 and 10 min, in the presence/absence of oligomycin A, a well-known specific inhibitor of mitochondrial ATP synthase or carbonyl cyanide 4-(trifluoromethoxy) phenylhydrazone (FCCP), a potent disruptor of the electron chain function. Therefore, this work makes a step towards understanding the action of red light on, specifically, sperm mitochondria and, hence, on the overall sperm function, by investigating the red-light action on the electron chain. Our hypothesis is that the use of these two agents should disrupt electron chain function and abolish the stimulating effects of red-light upon mitochondrial function when they block the crucial, involved part of the electron chain.

## 2. Materials and Methods

### 2.1. Semen Samples

Semen samples were provided by a commercial farm (Servicios Genéticos Porcinos, S.L. Roda de Ter, Spain) and came from 17 separate boars. Animals were post-pubertal (2–3 years old), healthy and belonged to the Piétrain breed. Boar studs were housed in strictly climate-controlled conditions, fed with a standard diet and provided with water ad libitum. Semen was collected manually through the gloved-hand method, and the obtained sperm-rich fractions were immediately diluted at 17 °C to a final concentration of 2 × 10^7^ sperm/mL in a commercial extender (Duragen^®^, Magapor, S.L.; Ejea de los Caballeros, Zaragoza, Spain). Diluted semen was split into 90 mL commercial AI doses, and two doses of each ejaculate were transported at 17 °C to our laboratory for 60 min.

### 2.2. Ethics

Pig ejaculates utilized in this study were not collected for the unique purpose of investigation, but for their commercial use. Thus, the farm provided us with some doses, and the remaining ones were used for AI. Therefore, there was no need for any specific ethical approval to perform this work, as no animal was manipulated by the authors.

### 2.3. Experimental Design

Upon arrival, samples were confirmed to fulfill quality standards, which were ≥80% membrane-intact spermatozoa (SYBR14^+^/PI^−^), ≥85% morphologically normal spermatozoa and ≥70% total motile spermatozoa. Following this, each semen sample was split into separate 1.5 mL aliquots that were subjected to three red-light irradiation protocols (LED, 620–630 nm; PhastBlue^®^, IUL, S.L.; Barcelona, Spain). These three protocols consisted of exposing sperm to red-light for 1 (1′), 5 (5′) or 10 min (10′). In all cases, the temperature within the PhastBlue^®^ system was maintained at 20 °C. Controls consisted of 1.5 mL tubes kept at 20 °C in the dark for 10 min. In addition to the aforementioned, semen samples were also exposed to the same three protocols (i.e., 1′, 5′ and 10′) or non-exposed (control) in the presence of either 5 µM oligomycin A or 5 µM FCCP. Both agents were added to semen samples 10 min prior to exposure to red-light. These two concentrations were chosen based on previous works [25,26,27,28,29] and preliminary, dose–response experiments and corresponded to those that showed a complete inhibitory effect without causing toxicity, which was based on sperm viability and acrosome integrity data.

Following irradiation, samples were used to evaluate sperm motility through a computer-assisted sperm analysis system (CASA); sperm membrane and acrosome integrity, mitochondrial membrane potential, and intracellular ROS and calcium levels by flow cytometry; O_2_ consumption; intracellular ATP levels; and total cytochrome *c* oxidase (CCO) activity. In the case of intracellular ATP levels and total CCO activity, samples were first centrifuged at 2000× *g* and 20 °C for 30 s, and the resulting pellet was frozen by plunging samples into liquid N_2_. Samples were stored at −80 °C until use.

### 2.4. Sperm Motility

Sperm motility was determined through a CASA system (Integrated Sperm Analysis System V1.0; Proiser; Valencia, Spain), based on the settings and parameters described previously [30]. The following kinematic parameters were evaluated: curvilinear velocity (VCL), straight line velocity (VSL), average pathway velocity (VAP), linearity coefficient (LIN), straightness coefficient (STR), wobble coefficient (WOB), amplitude of lateral head displacement (mALH), beat cross frequency (BCF), dance (DNC, VCL × ALH), absolute mean angular displacement (absMAD) and algebraic mean angular displacement (algMAD). Briefly, samples were warmed at 38 °C for 5 min in a water bath prior to placing 6 µL into a 20-micron Leja^®^ Standard Count Chamber Slide (Leja Products B.V.; Nieuw Vennep, The Netherlands). Three replicates of 1000 sperm each were evaluated before calculating the corresponding mean ± standard error of the mean (SEM). Total motility was defined as the percentage of sperm with VAP ≥ 10 µm/s, whereas progressive motility was considered to be the percentage of motile sperm with a STR ≥ 45%. Individual kinematic parameters (VSL, VCL, VAP, LIN, STR and BCF) were used to determine sperm motile subpopulations.

### 2.5. Flow Cytometry

Flow cytometry was used to determine plasma membrane and acrosome integrity, mitochondrial membrane potential (MMP), and intracellular levels of superoxides, peroxides and calcium, following the recommendations of the International Society for Advancement of Cytometry [31]. In all analyses, sperm concentration was previously adjusted to 1 × 10^6^ spermatozoa/mL in a final volume of 500 µL with Beltsville Thawing Solution (BTS), and three technical replicates were evaluated. Samples were examined using a Cell Laboratory QuantaSC cytometer (Beckman Coulter, Fullerton, CA, USA), and the sheath flow rate was set at 4.17 µL/min. Electronic volume (EV) and side scatter (SS) were recorded in a log-linear mode (EV/SS dot plots) for 10,000 events per replicate. The analyzer threshold was adjusted on the EV channel to exclude subcellular debris (particle diameter < 7 µm) and cell aggregates (particle diameter > 12 µm), and compensation was used to minimize fluorescence spill-over into a different channel. Information on all events was collected with List-mode Data files (EV, SS, FL1, FL2 and FL3) and processed using the Cell Lab QuantaSC MPL Analysis Software (version 1.0; Beckman Coulter). In all assessments, data were corrected using the procedure described by Petrunkina et al. [32], based on the percentage of debris particles (SYBR14^−^/PI^−^) determined through SYBR14/PI staining. Fluorochromes were purchased from Molecular Probes^®^ (Invitrogen^®^, Thermo Fisher Scientific, Waltham, MA, USA) and diluted with dimethyl sulfoxide (DMSO).

#### 2.5.1. Plasma Membrane Integrity

Percentages of membrane-intact spermatozoa were determined using the LIVE/DEAD^®^ Sperm Viability Kit (SYBR14/PI; [33]). Three separate sperm populations were identified. One of these populations corresponded to membrane-intact spermatozoa (SYBR14^+^/PI^−^), whereas the other two subpopulations (SYBR14^−^/PI^+^ and SYBR14^+^/PI^+^) corresponded to spermatozoa with different degrees of plasma membrane alterations. Debris and non-sperm particles were detected as SYBR14^−^/PI^−^, and were not taken into account to calculate the percentages of the three sperm populations. Single-stained samples were used to set EV gain, FL1 and FL3 PMT-voltages and for compensation of FL1 spill-over into the FL3-channel (2.45%).

#### 2.5.2. Acrosome Integrity

Acrosome integrity was determined through staining with peanut agglutinin from *Arachis hypogaea* conjugated with fluorescein isothiocyanate (FITC-PNA) and ethidium homodimer (EthD-1), following the protocol set by Cooper and Yeung [34] and adapted to pig spermatozoa by Rocco et al. [35]. Following this procedure, four sperm populations were identified: (i) viable spermatozoa with an intact acrosome membrane (PNA-FITC^+^/EthD-1^−^); (ii) viable spermatozoa with a non-intact acrosome membrane (PNA-FITC^-^/EthD-1^−^); (iii) non-viable spermatozoa with an intact acrosome membrane (PNA-FITC^+^/EthD-1^+^); and (iv) non-viable spermatozoa with a non-intact acrosome membrane (PNA-FITC^−^/EthD-1^+^). Total percentages of spermatozoa with a non-intact acrosome membrane resulted from summing PNA-FITC^−^/EthD-1^−^ and PNA-FITC^−^/EthD-1^+^ populations. FL1 spill-over into the FL3 channel (2.70%) was compensated.

#### 2.5.3. Mitochondrial Membrane Potential

Mitochondrial membrane potential (MMP) was evaluated through staining with JC1 (5,5′,6,6′-tetrachloro-1,1′,3,3′tetraethylbenzimidazolylcarbocyanine iodide; final concentration: 0.3 µM) [36]. Three sperm populations were distinguished: (i) spermatozoa with high MMP, characterized by the formation of JC1 aggregates (JC1_agg_), which exhibited orange staining; (ii) spermatozoa with intermediate/medium MMP, characterized by mitochondria that were stained in both orange and green; and (iii) spermatozoa with low MMP, characterized by the presence of JC1 monomers (JC1_mon_), which emitted green fluorescence. Results are shown as percentages of spermatozoa with high MMP and intermediate/medium MMP, and as the ratios between the geometric mean of fluorescence intensities (GMFI) of JC1_agg_ (FL2; orange) and JC1_mon_ (FL1; green) for the sperm populations with high and intermediate/medium MMP (JC1_agg_/JC1_mon_ ratio—i.e., orange/green ratio).

#### 2.5.4. Intracellular Levels of Superoxide and Peroxide

Intracellular levels of superoxide (O_2_^−^) radicals were analyzed through staining with hydroethidine and YO-PRO-1 (HE/YO-PRO-1) fluorochromes, whereas peroxide (H_2_O_2_) radicals were determined through staining with 2′-7′-dichlorodihydrofluorescein diacetate and propidium iodide (H_2_DCFDA/PI). Both tests were performed as described in Guthrie and Welch [37], and results are shown as percentages of viable spermatozoa with high superoxide levels (E^+^/YO-PRO-1^−^) and of viable spermatozoa with high peroxide levels (DCF^+^/PI^−^). In the case of HE/YO-PRO-1, FL3 spill-over into the FL1-channel was compensated (5.06%), and in that of H_2_DCFDA/PI, FL1 spill-over into the FL3-channel was also compensated (2.45%). Results are shown as percentages of spermatozoa with high levels of peroxides and superoxides within the viable sperm population.

#### 2.5.5. Intracellular Calcium Levels

Previous studies found that Fluo3 mainly stains mitochondrial calcium in pig sperm [38]. For this reason, we combined this fluorochrome with propidium iodide (Fluo3/PI), as described by Kadirvel et al. [39], and the following four populations were identified: (i) viable spermatozoa with low levels of intracellular calcium (Fluo3^−^/PI^−^); (ii) viable spermatozoa with high levels of intracellular calcium (Fluo3^+^/PI^−^); (iii) non-viable spermatozoa with low levels of intracellular calcium (Fluo3^−^/PI^+^); and (iv) non-viable spermatozoa with high levels of intracellular calcium (Fluo3^+^/PI^+^). FL1 spill-over into the FL3-channel (2.45%) and FL3 spill-over into the FL1-channel (28.72%) were compensated. Results are shown as percentages of spermatozoa with high intracellular calcium levels within the viable sperm population, and as GMFI of Fluo3.

### 2.6. Determination of JC1-Staining Witch Laser Confocal Laser Microscopy

Spermatozoa stained with JC1 were also analyzed by confocal laser scanning microscopy to detect the specific localization of mitochondria with high membrane potential (stained in orange) in the mid-piece. With this purpose, sperm were incubated at the same conditions to those used for flow cytometry—i.e., 38 °C for 30 min (final concentration of JC1: 0.3 µM). In order to avoid an excessive motility constraint that could alter JC1-staining along the mid-piece, 20 µL of each sample was placed onto the bottom of a well (4-well plates). Samples were observed under a Leica TCS 4D confocal laser scanning microscope (Leica lasetechnik; Wertrieb, Germany) adapted to an inverted Leitz DMIRBE microscope at 63× (N.4 oil; Plan-Apo Lens; Leitz; Stuttgart, Germany). The light source was an argon/krypton laser (74 mW), and fluorescence detection was performed through an excitation wavelength of 488 nm. Depending on how mitochondria were stained, two emission wavelengths were detected. The first emission wavelength corresponded to low MMP mitochondria (JC1_mon_), which emitted at 530 nm (green), whereas the second one corresponded to high MMP mitochondria (JC1_agg_), which emitted at 590 nm (orange). Since JC1-stained sperm maintained their motility, a sequential track of images per sample was taken at 38 °C for 20 s (rate: one capture/s).

### 2.7. Determination of Intracellular ATP Levels

Intracellular ATP levels were determined following the protocol set by Chida et al. [40]. Briefly, 1 mL semen aliquots were centrifuged at 17 °C for 30 s after light-stimulation, and pellets were immediately plunged into liquid N_2_. Frozen pellets were subsequently stored at −80 °C for three weeks. Thereafter, pellets were resuspended in 300 µL ice-cold 10 mM 2-[4-(2-hydroxyethyl)piperazin-1-yl]ethanesulfonic acid (HEPES) buffer containing 250 mM sucrose (pH was adjusted to 7.4). Resuspended pellets were sonicated (10 kHz, 20 pulses; Bandelin Sonopuls HD 2070; Bandelin Electronic GmbH and Co., Berlin, Germany), while tubes were kept on ice to avoid specimen heating. Samples were subsequently centrifuged at 1000× *g* and 4 °C for 10 min and supernatants were collected. Twenty µL was used to determine total protein content, and the remaining volume was mixed with 300 µL ice-cold 10% (v:v) trichloroacetic acid and kept at 4 °C for 20 s. Samples were subsequently centrifuged at 1000× *g* and 4 °C for 30 s, and supernatants were carefully separated from the pellet and again centrifuged at 1000× *g* and 4 °C for 10 min. Resulting supernatants were mixed with two volumes of 1 M Tris-acetate buffer (p.75), and ATP content was determined in these final suspensions using the Invitrogen^®^ ATP Determination Kit (ThermoFisher Bioscientific; Waltham, MA, USA; catalogue number: A22066), following the manufacturer’s instructions. Determinations of ATP content were carried out through an Infinite F200 fluorimeter (TECAN^®^), using 96-wells microplates for fluorescence-based assays (Invitrogen^®^). To normalize data, total protein of samples was determined through the Bradford method [41], by using a commercial kit (Bio-Rad laboratories; Hercules, CA, USA).

### 2.8. Determination of O_2_ Consumption Rate

Determination of O_2_ consumption rate was performed using a SensorDish^®^ Reader (SDR) system (PreSens Gmbh; Regensburg, Germany). Semen aliquots of 1 mL, previously exposed to red-light (or not irradiated in the case of controls), were transferred onto Oxodish^®^ OD24 plates (24 wells/plate) specifically designed for this device. Plates were sealed with Parafilm^®^, introduced into the SDR system, and incubated at 37 °C (controlled atmosphere) for 2 h. During that time, O_2_ concentration was recorded in each well at a rate of one reading per min. Data were exported to an Excel file (see Supplementary Figure S1 for representative curves), and final O_2_ consumption rate was normalized against the total number of viable spermatozoa per sample, which was determined through flow cytometry (SYBR14^+^/PI^−^ spermatozoa) using another aliquot.

### 2.9. Determination of Cytochrome C Oxidase Activity

Activity of cytochrome *c* oxidase (CCO) was determined in mitochondria-enriched sperm fractions, as described elsewhere [42]. Briefly, 1 mL sperm aliquots, previously irradiated with red-light (or not irradiated in the case of controls), were centrifuged at 1000× *g* and 17 °C for 30 s. The resulting pellets were immediately plunged into liquid N_2_ and stored for three weeks. Pellets were resuspended in 500 µL ice-cold PBS and sonicated (10 kHz, 20 pulses; Bandelin Sonopuls HD 2070). Thereafter, 500 µL Percoll^®^ at a concentration of 1.055 mg/mL in PBS at 4 °C was placed onto each sperm homogenate. Samples were centrifuged at 3000× *g* and 10 °C for 45 min, and the mitochondria-enriched fraction was carefully harvested with a micropipette and transferred into a new 1.5 mL tube. Samples were again centrifuged at 12,000× *g* and 20 °C for 2 min, and the resulting pellets were resuspended in 100 µL PBS at 20 °C. These mitochondria-enriched suspensions were split into two separate aliquots. The first one was used to determine CCO activity using a commercial kit (Cytochrome *c* Oxidase Assay Kit; Sigma-Aldrich; catalogue number CYTOCOX1). The other aliquot (10 µL) was used to determine total protein content through a commercial kit based on the Bradford method (Bio-Rad) [41]. Enzyme activity was normalized against the total protein content.

### 2.10. Statistical Analyses

Statistical analyses were conducted using a statistical package (SPSS^®^ Ver. 25.0 for Windows; IBM corp., Armonk, NY, USA). Data were first tested for normality and homogeneity of variances through Shapiro–Wilk and Levene tests, respectively. When required, data were transformed through arcsin √x. The effects of red-light stimulation patterns on motility parameters, percentages of spermatozoa with an intact plasma membrane (SYBR14^+^/PI^−^), spermatozoa with an intact acrosome (PNA-FITC^+^), spermatozoa with high MMP (JC1_agg_^+^), viable spermatozoa with high intracellular calcium levels (Fluo3^+^/PI^−^), viable spermatozoa with high superoxide levels (E^+^/YO-PRO-1^−^) and viable spermatozoa with high peroxide levels (DCF^+^/PI^−^); geometric mean fluorescence intensity (GMFI) of JC1_agg_^+^, Fluo3^+^, E^+^ and DCF^+^; intracellular levels of ATP; O_2_ consumption rate; and cytochrome *c* oxidase activity were evaluated through a two-way analysis of variance (ANOVA) followed by a post-hoc Sidak test. In this model, factors were: (a) irradiation pattern (i.e., non-irradiation, irradiation for 1 min, irradiation for 5 min and irradiation for 10 min) and (b) presence/absence of disruptors (control, oligomycin A and FCCP).

Motile sperm subpopulations were determined through the protocol described in Luna et al. [43]. In brief, individual kinematic parameters (VCL, VSL, VAP, LIN, STR, WOB, ALH and BCF) recorded for each sperm cell were used as independent variables in a Principal Component Analysis (PCA). Kinematic parameters were sorted into PCA components, and the obtained matrix was subsequently rotated using the Varimax method with Kaiser normalization. As a result, each spermatozoon was assigned regression scores for the new PCA components and these values were subsequently used to run a two-step cluster analysis based on the log-likelihood distance and the Schwarz’s Bayesian Criterion. Three sperm subpopulations were identified, and each individual spermatozoon was clustered into one of these three subpopulations (SP1, SP2 or SP3). Thereafter, percentages of spermatozoa belonging to each subpopulation were calculated per sample, and were further used to evaluate the effects of red-light stimulation on the distribution of motile sperm subpopulations through a two-way ANOVA followed by Sidak post-hoc test. Factors were: (a) irradiation pattern (i.e., non-irradiation, irradiation for 1 min, irradiation for 5 min and irradiation for 10 min) and (b) presence/absence of disruptors (control, oligomycin A and FCCP).

In all analyses, the level of significance was set at *p ≤* 0.05. Data are shown as the mean ± standard error of the mean (SEM).

## 3. Results

### 3.1. Effects of Red-Light Stimulation on Plasma Membrane and Acrosome Integrity in the Presence or Absence of Either Oligomycin A or FCCP

**Figure 1:**
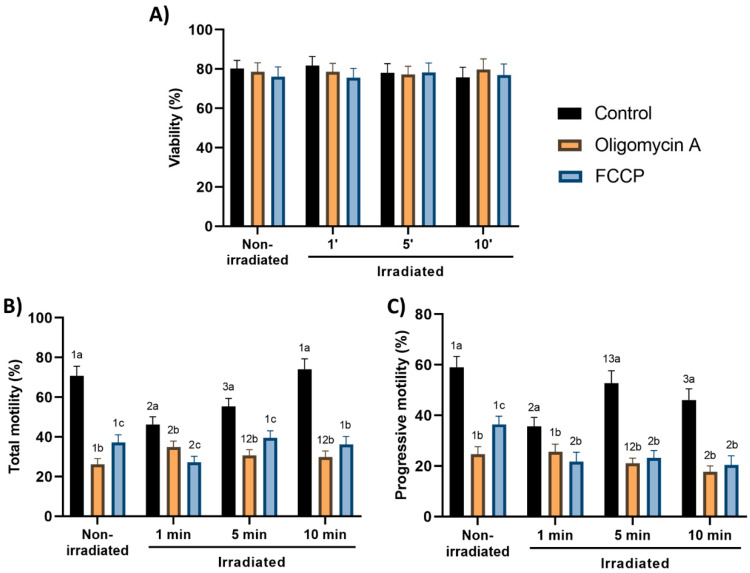
Effects of light-stimulation on percentages of membrane-intact spermatozoa (SYBR14^+^/PI^−^; (**A**)), total (**B**) and progressive (**C**) sperm motility in the presence/absence of either oligomycin A or FCCP. Sperm were not irradiated (non-irradiated), or irradiated for 1, 5 and 10 min. Control: sperm not incubated with either oligomycin A or FCCP. Oligomycin A: sperm incubated with 5 µM oligomycin A. FCCP: sperm incubated with 5 µM FCCP. Different superscript numbers (*1–3*) mean significant (*p* < 0.05) differences between light-stimulation patterns within a given treatment (i.e., control, oligomycin A or FCCP). Different superscript letters (*a–c*) indicate significant (*p* < 0.05) differences between treatments within a given irradiation pattern (non-irradiated, light-stimulation for 1 min, light-stimulation for 5 min or light-stimulation for 10 min). Results are expressed as mean ± SEM for seven separate experiments.

No red-light stimulation protocol had any effect on the percentages of membrane-intact spermatozoa (SYBR14^+^/PI^−^) when compared to non-irradiated samples (80.3 ± 4.1%; Figure 1A). Similarly, no protocol induced any change on the percentage of acrosome-intact spermatozoa when compared to non-irradiated samples (control, non-irradiated sperm: 18.7 ± 3.5%; Supplementary Figure S2).

### 3.2. Effects of Red-Light Stimulation on Sperm Motility in the Presence or Absence of Either Oligomycin A or FCCP

Percentages of total motile spermatozoa in the presence of oligomycin A (26.1 ± 2.9%) and FCCP (37.1 ± 3.8%) were significantly (*p* < 0.05) lower than those of the control (70.8 ± 4.7%; Figure 1B). These differences were also observed in the percentage of progressively motile spermatozoa (Figure 1C). Moreover, light-stimulation altered total and progressive sperm motility, but the impact differed between the three protocols. Thus, whereas light irradiation of sperm without the presence of either oligomycin A or FCCP for 1 and 5 min significantly (*p* < 0.05) decreased total sperm motility compared to non-irradiated sperm, it did not make such an impact when samples were irradiated for 10 min (Figure 1B). Moreover, in the absence of oligomycin A and FCCP, percentages of progressively motile spermatozoa were significantly (*p* < 0.05) lower after irradiation for 1 and 10 min than in non-irradiated sperm, but they did not differ between the 5 min protocol and non-irradiated samples (Figure 1C). The presence of oligomycin A and FCCP during light-stimulation led to a significant reduction in both total and progressive sperm motilities when compared to their respective control samples (Figure 1B,C).

With regard to the effects of light-stimulation on sperm kinematic parameters, irradiating control samples (i.e., without oligomycin A or FCCP) for 1 min led to a significant (*p* < 0.05) decrease in VCL, VSL and VAP; in contrast, the other two light-stimulation protocols (5 min and 10 min) had a much lower impact on these parameters (Table 1). On the other hand, the presence of either oligomycin A or FCCP also exerted a negative effect on sperm kinematic parameters, regardless of whether or not sperm were exposed to red-light. This was particularly apparent in the case of VCL and DNC; however, whereas the presence of oligomycin A or FCCP had less effect following light-stimulation for 1 min, the extent of that reduction was higher when sperm were irradiated for 5 and 10 min (Table 1).

**Figure 2:**
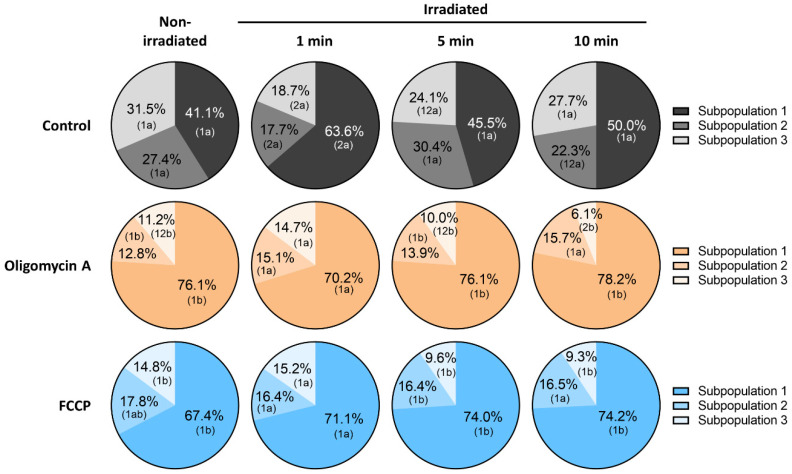
Effects of light-stimulation on the motile sperm subpopulation structure in the presence/absence of either oligomycin A or FCCP. Results are shown as % of motile spermatozoa belonging to each subpopulation in three rows, as follows: Control, sperm incubated without either oligomycin A or FCCP; Oligomycin A, sperm incubated with 5 µM oligomycin A; and FCCP, sperm incubated with 5 µM FCCP. Data are also split into four columns as follows: non-irradiated: non-irradiated sperm; 1 min: sperm irradiated for 1 min; 5 min: sperm irradiated for 5 min; 10 min: sperm irradiated for 10 min. Different superscript numbers (*1*, *2*) mean significant (*p* < 0.05) differences between light-stimulation patterns within a given treatment (i.e., control, oligomycin A or FCCP). Different superscript letters (*a*, *b*) indicate significant (*p* < 0.05) differences between treatments within a given irradiation pattern (non-irradiated, light-stimulation for 1 min, light-stimulation for 5 min or light-stimulation for 10 min). Results are expressed as mean ± SEM for seven separate experiments.

Finally, the percentages of spermatozoa belonging to each motile sperm subpopulation changed following light-stimulation, and in response to the presence of oligomycin A or FCCP. Although three motile sperm subpopulations were identified in all treatments/irradiation protocols, stimulation of sperm with red-light for 1 min significantly (*p* < 0.05) decreased the percentages of Subpopulation 3, which was the one that exhibited the highest VSL, LIN and STR (Supplementary Table S1). The presence of either oligomycin A or FCCP did not alter the structure of sperm subpopulations observed after irradiating spermatozoa for 1 min. Moreover, percentages of spermatozoa belonging to each subpopulation following light-irradiation for 5 and 10 min did not differ from non-irradiated samples (Figure 2). Interestingly, the presence of oligomycin A or FCCP significantly (*p* < 0.05) decreased the percentages of spermatozoa belonging to Subpopulation 3 in non-irradiated samples and in two light-stimulation protocols (5 and 10 min), with a concomitant increase in the percentage of cells belonging to Subpopulation 1.

### 3.3. Effects of Red-Light Stimulation on Mitochondrial Membrane Potential in the Presence or Absence of Either Oligomycin A or FCCP

**Figure 3:**
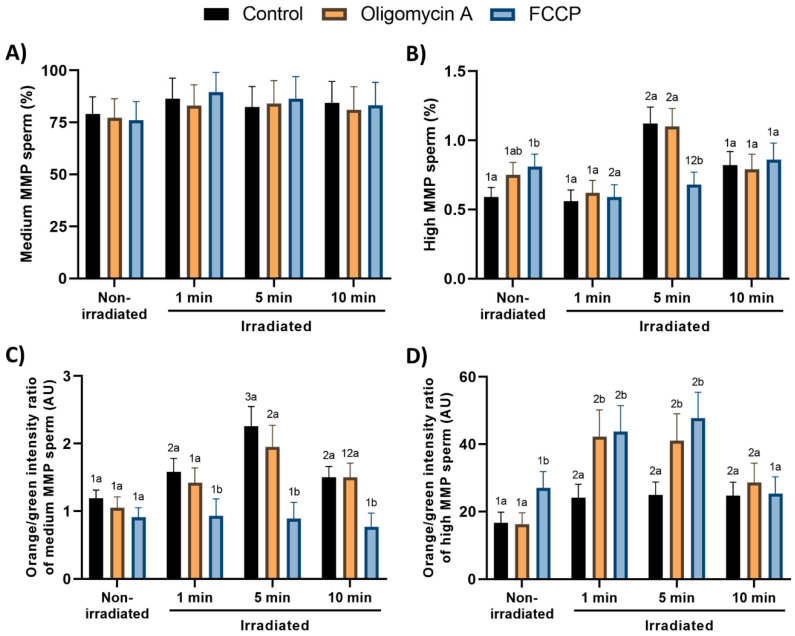

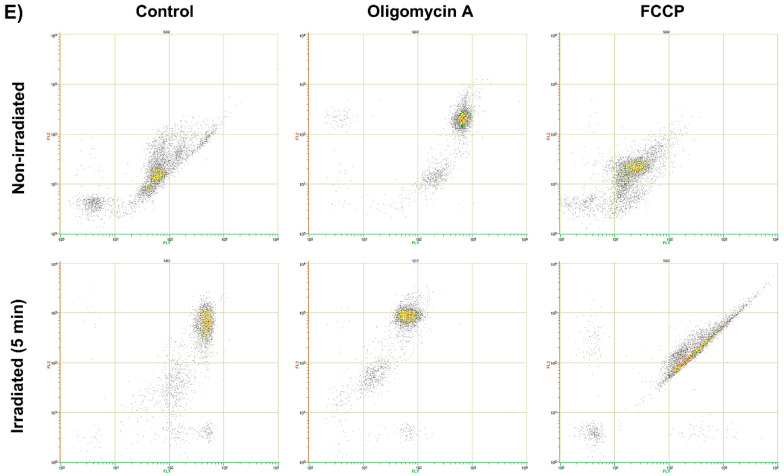
Effects of light-stimulation on mitochondrial membrane potential in the presence/absence of either oligomycin A or FCCP. Results are shown as percentages of spermatozoa with intermediate (medium) mitochondrial membrane potential (MMP) (**A**) and high MMP (**B**), and as JC1_agg_/JC1_mon_ (orange:green intensity) ratios of intermediate-MMP (**C**) and high-MMP sperm populations (**D**). Sperm were not irradiated (non-irradiated), or irradiated for 1, 5 and 10 min. Representative dot-plot graphs for non-irradiated samples and samples irradiated for 5 min are also shown (**E**). Control: sperm not incubated with either oligomycin A or FCCP. Oligomycin A: sperm incubated with 5 µM oligomycin A. FCCP: sperm incubated with 5 µM FCCP. Different superscript numbers (*1–3*) mean significant (*p* < 0.05) differences between light-stimulation patterns within a given treatment (i.e., control, oligomycin A or FCCP). Different superscript letters (*a*, *b*) indicate significant (*p* < 0.05) differences between treatments within a given irradiation pattern (non-irradiated, light-stimulation for 1 min, light-stimulation for 5 min or light-stimulation for 10 min. Results are expressed as mean ± SEM for seven separate experiments.

Percentages of spermatozoa with high MMP and JC1_agg_/JC1_mon_ (orange/green intensity) ratio of the sperm population with high MMP were low (0.59 ± 0.07%; 16.7 arbitrary units ± 3.1 arbitrary units; Figure 3B,D) in control, non-irradiated samples. While the addition of oligomycin A did not modify these parameters, that of FCCP induced a significant (*p* < 0.05) increase in both the percentage and JC1_agg_/JC1_mon_ ratio of the sperm population with high MMP (Figure 3B,D,E; Supplementary Figure S3).

On the other hand, light-stimulation for 5 min induced a significant (*p* < 0.05) increase in the percentage of spermatozoa with high MMP in both control samples and those treated with oligomycin A when compared with their respective non-irradiated counterparts. The presence of FCCP, however, counteracted that increase, the percentages of spermatozoa with high MMP being similar to those of non-irradiated samples. In addition, all light-stimulation protocols, except when sperm were irradiated in the presence of FCCP, significantly (*p* < 0.05) increased JC1_agg_/JC1_mon_ ratios in the sperm population with high MMP (e.g., 1 min: 24.1 arbitrary units ± 4.0 arbitrary units vs. control: 16.7 arbitrary units ± 3.1 arbitrary units; Figure 3D). While both oligomycin A and FCCP significantly (*p* < 0.05) increased the JC1_agg_/JC1_mon_ ratio in the sperm population with high MMP following light-stimulation for 1 and 5 min (compared with the respective irradiated controls), this effect was not observed when samples were irradiated for 10 min (Figure 3D).

In all treatments, percentages of spermatozoa with intermediate MMP were much higher than those of spermatozoa with high MMP; the JC1_agg_/JC1_mon_ ratio in this intermediate MMP population was lower than that of the high-MMP one (Figure 3A,C; Supplementary Figure S3). Whereas percentages of spermatozoa exhibiting intermediate MMP were not affected by light-stimulation protocols and the presence of either oligomycin A or FCCP, the JC1_agg_/JC1_mon_ ratio in this sperm population was significantly (*p* < 0.05) increased in light-stimulated spermatozoa, especially after irradiating sperm for 5 min (Figure 3C). Moreover, although the presence of oligomycin A did not alter the hike observed in the JC1_agg_/JC1_mon_ ratio after light-stimulation, FCCP counteracted that increase in the three irradiation protocols (Figure 3C).

**Figure 4:**
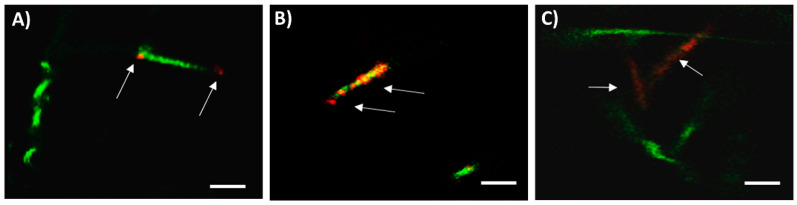
Specific localization of mitochondria with high mitochondrial membrane potential (JC1 staining) in sperm incubated in the presence/absence of either oligomycin A or FCCP. Representative video images of laser confocal microscope for control (**A**), oligomycin A (**B**) and FCCP (**C**). Arrows indicate the localization of mitochondria with high MMP (orange staining). The appearance of sashed mid-pieces is due to the frame rate of the laser confocal microscope, which was lower than sperm velocity. Images are representative for seven separate experiments. Bar size: 10 µm.

It is worth mentioning that the specific localization of orange-stained mitochondria (corresponding to spermatozoa with high MMP) differed between control and oligomycin A- and FCCP-treated samples. Thus, whereas orange-stained mitochondria in the absence of these two disruptors were found to be confined to the two mid-piece poles (Figure 4A and Supplementary File S1), sperm treated with oligomycin A showed a distinct pattern, with orange-stained mitochondria being distributed along the entire mid-piece (Figure 4B and Supplementary File S2). Sperm treated with FCCP showed faint but uniform orange-staining pattern along the mid-piece (Figure 4C and Supplementary File S3). No light-stimulation pattern changed the distribution of orange-stained mitochondria along the mid-piece.

### 3.4. Effects of Red-Light Stimulation on Superoxide and Peroxide Levels in the Presence or Absence of Either Oligomycin A or FCCP

**Figure 5:**
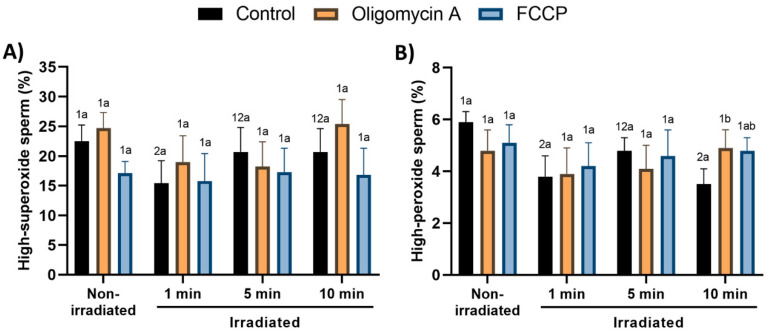
Effects of light-stimulation on intracellular levels of superoxides (**A**) and peroxides (**B**) in the presence/absence of either oligomycin A or FCCP (results are shown as percentages of spermatozoa with high levels of peroxides and superoxides within the viable sperm population). Sperm were not irradiated (non-irradiated), or irradiated for 1, 5 and 10 min. Control: sperm not incubated with either oligomycin A or FCCP. Oligomycin A: sperm incubated with 5 µM oligomycin A. FCCP: sperm incubated with 5 µM FCCP. Different superscript numbers (*1*, *2*) mean significant (*p* < 0.05) differences between light-stimulation patterns within a given treatment (i.e., control, oligomycin A or FCCP). Different superscript letters (*a*, *b*) indicate significant (*p* < 0.05) differences between treatments within a given irradiation pattern (non-irradiated, light-stimulation for 1 min, light-stimulation for 5 min or light-stimulation for 10 min. Results are expressed as mean ± SEM for seven separate experiments.

With regard to control samples, percentages of spermatozoa with high intracellular superoxide levels (within the viable sperm population) were significantly (*p* < 0.05) lower in samples irradiated for 1 min than in those non-irradiated or irradiated for 5 or 10 min (Figure 5A; Supplementary Figure S4). Neither oligomycin A nor FCCP affected the impact of light-stimulation on the percentages of viable spermatozoa with high intracellular superoxide levels.

Light-stimulation, especially for 1 and 10 min, significantly (*p* < 0.05) decreased the proportions of viable spermatozoa with high peroxide levels (Figure 5B; Supplementary Figure S5). While this decrease was not altered by the presence of either oligomycin A or FCCP following irradiation for 1 and 5 min, oligomycin A, but not FCCP, significantly counteracted the reduction observed when samples were irradiated for 10 min. Finally, non-irradiated spermatozoa did not show significant differences between the control and oligomycin A- and FCCP-treated samples (Figure 5B).

### 3.5. Effects of Red-Light Stimulation on Intracellular Calcium Levels in the Presence or Absence of Either Oligomycin A or FCCP

**Figure 6:**
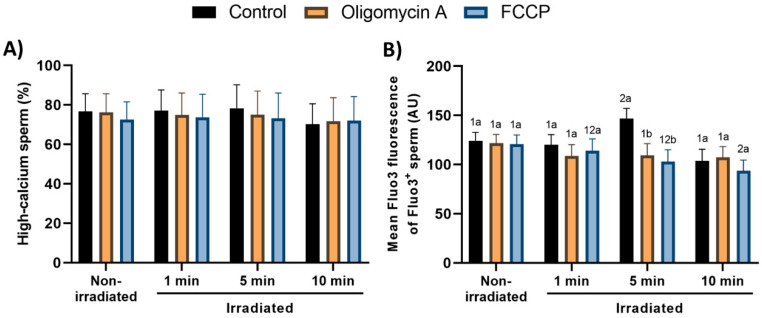
Effects of light-stimulation on percentages of spermatozoa with high intracellular calcium levels (within the viable sperm population; (**A**)) and geometric mean intensity of Fluo3^+^ (**B**) in the presence/absence of either oligomycin A or FCCP. Sperm were not irradiated (non-irradiated), or irradiated for 1, 5 and 10 min. Control: sperm not incubated with either oligomycin A or FCCP. Oligomycin A: sperm incubated with 5 µM oligomycin A. FCCP: sperm incubated with 5 µM FCCP. Different superscript numbers (*1*, *2*) mean significant (*p* < 0.05) differences between light-stimulation patterns within a given treatment (i.e., control, oligomycin A or FCCP). Different superscript letters (*a*, *b*) indicate significant (*p* < 0.05) differences between treatments within a given irradiation pattern (non-irradiated, light-stimulation for 1 min, light-stimulation for 5 min or light-stimulation for 10 min. Results are expressed as mean ± SEM for seven separate experiments.

Percentages of viable spermatozoa with high intracellular calcium levels did not differ amongst non-irradiated samples (i.e., control vs. oligomycin A and FCCP; Figure 6A and Supplementary Figure S6). However, light-stimulation for 5 min significantly (*p* < 0.05) augmented the geometric mean of fluorescence intensity of Fluo3^+^ in Fluo3^+^/PI^−^ sperm. This increase was counteracted by both oligomycin A and FCCP (Figure 6B).

### 3.6. Effects of Red-Light Stimulation on Intracellular Levels of ATP and Oxygen Consumption in the Presence or Absence of Either Oligomycin A or FCCP

**Figure 7:**
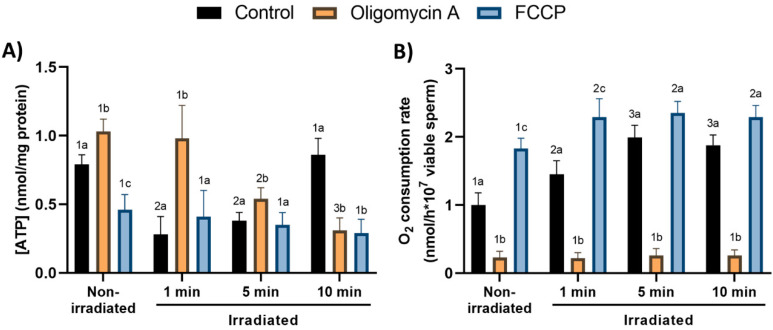
Effects of light-stimulation on intracellular ATP levels (**A**) and O_2_ consumption rate (**B**) in the presence/absence of either oligomycin A or FCCP. Sperm were not irradiated (non-irradiated), or irradiated for 1, 5 and 10 min. Control: sperm not incubated with either oligomycin A or FCCP. Oligomycin A: sperm incubated with 5 µM oligomycin A. FCCP: sperm incubated with 5 µM FCCP. Different superscript numbers (*1–3*) mean significant (*p* < 0.05) differences between light-stimulation patterns within a given treatment (i.e., control, oligomycin A or FCCP). Different superscript letters (*a–c*) indicate significant (*p* < 0.05) differences between treatments within a given irradiation pattern (non-irradiated, light-stimulation for 1 min, light-stimulation for 5 min or light-stimulation for 10 min. Results are expressed as mean ± SEM for seven separate experiments.

Control, non-irradiated spermatozoa showed low intracellular ATP levels (0.79 nmol/mg protein ± 0.07 nmol/mg protein; Figure 7A). These levels were significantly (*p <* 0.05) increased in the presence of oligomycin A, and significantly (*p <* 0.05) decreased in the presence of FCCP (Figure 7A). Light-stimulation for 1 and 5 min significantly (*p <* 0.05) decreased intracellular ATP levels, but such decreases were counteracted completely or partially by oligomycin A, when spermatozoa were irradiated for 1 or 5 min, respectively (Figure 7A). The presence of both oligomycin A and FCCP in sperm irradiated for 10 min induced a strong, significant (*p <* 0.05) decrease in ATP content when compared with control samples irradiated for 10 min (Figure 7A).

In non-irradiated sperm, O_2_ consumption rate was significantly (*p <* 0.05) decreased by oligomycin A, and increased by FCCP (Figure 7B). In the absence of either oligomycin A or FCCP, light-stimulation significantly (*p <* 0.05) increased O_2_ consumption rate in the three protocols (i.e., 1, 5 and 10 min; Figure 7B). It is worth noting that while the presence of oligomycin A in irradiated spermatozoa dramatically (*p <* 0.05) decreased O_2_ consumption rates, completely abolishing the effects of light, combining red-light exposure for 1 min and FCCP led to higher O_2_ consumption rates compared to those observed in the absence of FCCP (Figure 7B).

### 3.7. Effects of Red-Light Stimulation on Cytochrome C Oxidase (CCO) Activity in the Presence or Absence of Either Oligomycin A or FCCP

**Figure 8:**
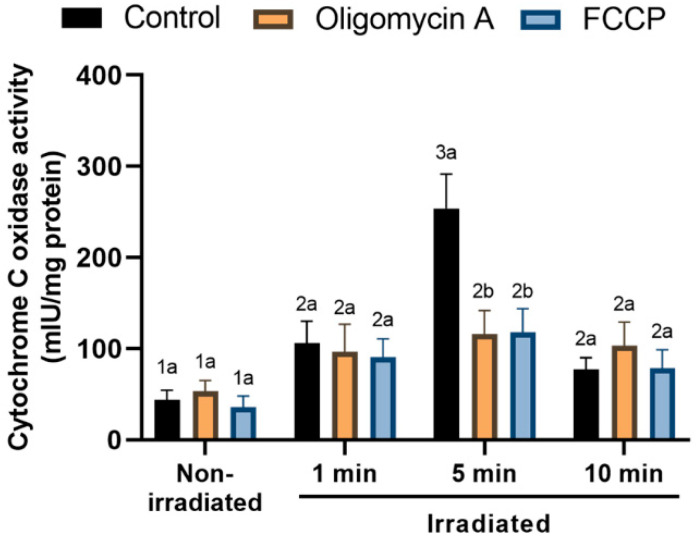
Effects of light-stimulation on cytochrome *c* oxidase activity in the presence/absence of either oligomycin A or FCCP. Sperm were not irradiated (non-irradiated), or irradiated for 1, 5 and 10 min. Control: sperm not incubated with either oligomycin A or FCCP. Oligomycin A: sperm incubated with 5 µM oligomycin A. FCCP: sperm incubated with 5 µM FCCP. Different superscript numbers (*1–3*) mean significant (*p* < 0.05) differences between light-stimulation patterns within a given treatment (i.e., control, oligomycin A or FCCP). Different superscript letters (*a*, *b*) indicate significant (*p* < 0.05) differences between treatments within a given irradiation pattern (non-irradiated, light-stimulation for 1 min, light-stimulation for 5 min or light-stimulation for 10 min. Results are expressed as mean ± SEM for seven separate experiments.

Non-irradiated spermatozoa showed low CCO activity (43.8 ± 10.8 mIU/mg protein; Figure 8), regardless of the presence of oligomycin A or FCCP. Light-stimulation induced a significant (*p* < 0.05) increase in CCO activity, which reached a peak after sperm were irradiated for 5 min (253.8 ± 37.6 mIU/mg protein; Figure 8). While neither oligomycin A nor FCCP affected the aforementioned increase when sperm were irradiated for 1 or 10 min, they both significantly (*p* < 0.05) counteracted the increase in CCO activity induced by the exposure of sperm to light for 5 min (Figure 8).

## 4. Discussion

The present study clearly indicates that the effects of red LED light on mammalian sperm function are related with changes in the mitochondrial electronic chain activity. Moreover, the impact of red-light appears to rely on the time of exposure, as indicated by the differences observed between protocols. Regarding the effects of red-light on the mitochondrial electronic chain, this could be explained through different hypothesis. The main one would be underpinned by the direct action on mitochondrial photosensitizers. In effect, crucial components of the electronic chain, such as cytochrome *c*, are sensitive to light at a wavelength range from 630 to 660 nm [24]. This sensitivity could explain our CCO activity results and would support the impact of red-light on mammalian sperm function. Indeed, while electron chain activity is usually related to energy production [42], it is also involved in the modulation of the overall eukaryotic cell function. In this respect, the electron chain plays a pivotal role in the production of reactive oxygen species (ROS), since mitochondria are the most important generating source of ROS in eukaryotic cells [44]. In addition, cytochrome complexes are also involved in the intrinsic apoptotic pathway [45], and both ROS generation and modulation of apoptotic-like changes are crucial in eliciting and controlling sperm capacitation [21]. Therefore, red light-induced changes in cytochrome *c* complex activity could ultimately affect how sperm capacitate and which their lifespan is.

Our results indicate that the presence of oligomycin A in non-irradiated sperm decreases O_2_ consumption, although this reduction is not concomitant with a complete inhibition of motility or a decrease in intracellular ATP levels. These results differ from others previously published in which oligomycin A did abolish motility without modifying either O_2_ consumption rate or ATP content [46]. These separate effects could be explained by differences between experimental conditions. In effect, whereas the study by Ramió-Lluch et al. [46] was performed under in vitro capacitation conditions, the current work was centered on semen stored in a commercial extender, which is specifically designed to maintain sperm activity/metabolism as low as possible. Related with this, it is worth mentioning that our results match with a recent study that used liquid-stored semen and tested the effects of oligomycin A and FCCP [47]. In the light of the aforementioned, one could suggest that the different conditions provided to sperm either by a capacitating medium or by a commercial extender would lead cells to be kept under a different functional status, which, in turn, would support separate reactivity against agents such as oligomycin A. In this context, it is worth bearing in mind that oligomycin A inhibits the activity of mitochondrial ATP synthase via binding its regulatory F0 subunit complex [48]. The F0 subunit complex modulates the proton flux that ultimately activates ATP synthase [49]. Since this means that the activity of the F0 subunit complex relies upon the precise proton flux, one could hypothesize that the inhibitory effect of oligomycin A could be different depending on that flux. This would imply that in conditions in which mitochondrial activity is very low or basal, such as those of this work, the effects of oligomycin A would be much less pronounced than in conditions with a stimulated mitochondrial activity, such as those of Ramió-Lluch et al. [46]. Regarding the results observed in both ATP content and sperm motility, Marin et al. [50] showed that when pig sperm are maintained in conditions similar to those of the current work, not only are ATP levels much lower than those of Ramió-Lluch et al. [46], but ATP is mainly produced via glycolysis. Thus, the combination of different ATP levels with separate glycolysis/mitochondria oxidative phosphorylation balance could be on the basis of the observed differences in both oligomycin A-sensitivity and specific motility parameters of sperm incubated in capacitating conditions or preserved at 17 °C.

Results observed for JC1-staining and motile sperm subpopulations suggest that the effects of red LED light on mammalian sperm are not homogeneous, but heavily rely upon the specific functional status of each individual spermatozoon. In fact, not only do the observed percentages of spermatozoa with high and intermediate MMP and their JC1_agg_/JC1_mon_ ratios support that the time of exposure to red-light has a different impact on mitochondrial membrane potential but also that not all spermatozoa exhibit the same response. Confocal microscope images corroborated this hypothesis, despite the distribution of JC1_agg_ and JC1_mon_ along the mitochondrial piece differing between the control and oligomycin A and FCCP treatments. In addition, the different response of each spermatozoon to red-light agrees with the presence of separate sperm populations based on JC1-staining [51], and also concur with our motile sperm subpopulations data. In this context, it is reasonable to assume that the different response to red LED light of each spermatozoon also induces variations in the motile subpopulation structure, in a similar fashion to when sperm are cryopreserved or subjected to in vitro capacitation [30,52,53].

Regarding the effects of light-stimulation and the presence of oligomycin A and FCCP on ROS levels, one should take into consideration two crucial aspects. The first is related to the low ROS levels found in pig spermatozoa under our experimental conditions. These low levels have already been reported [32,33,54] and have been related to the low metabolic rate of ejaculated, non-capacitated pig spermatozoa. In these conditions, pig sperm metabolism is mainly diverted to glycolysis rather than to mitochondrial oxidative phosphorylation [50], and sperm cells show low activity rates for their electron chain. Related with this, the second aspect that needs to be emphasized is that, under our experimental conditions, one should not expect much effect from any treatment/irradiation pattern on ROS levels, as we observed in this study. On the other hand, taking into account that, in all cases, ROS production should be low in our experimental conditions, the cell mechanisms devoted to the modulation of intracellular ROS levels, such as those related to glutathione, should be fully active in those sperm cells. Therefore, it is reasonable to suggest that the marginal impact on ROS levels under our experimental conditions together with the active mechanisms intended to control intracellular ROS levels could explain that the observed variations in intracellular peroxides and superoxides were so small.

Our results indicate that irradiating pig sperm with red-light induces, in all cases, an increase in O_2_ consumption rate, which was abolished by the addition of oligomycin A but was potentiated by FCCP, especially when sperm cells were light-stimulated for 1 min. As far as the effects of oligomycin A are concerned, it is worth mentioning that previous studies reported that pig sperm incubated in a capacitating medium containing that inhibitor do not show a decrease in O_2_ consumption rates [41]. This apparent discrepancy could be linked to the fact that, compared with sperm cells subjected to the activation induced by capacitating media, the metabolic conditions and hence mitochondrial activity of pig sperm are completely different when cells are incubated in a basal medium that, as the one used herein, maintains very low metabolic rates. In effect, while activated sperm, such as those tested in previous studies [41], would have mechanism/s that maintain the rhythm of the Krebs cycle regardless of the ATP synthase activity rate, non-capacitated sperm would maintain a close relationship between the activity of the Krebs cycle and ATP synthase. In this context, we can only speculate that one possible explanation for these differences could be related to mitochondrial uncoupling proteins. Thus, this issue warrants further research, including experiments addressing the role of mitochondrial activity in the control of pig sperm capacitation.

Still related to O_2_ consumption rates, and as aforementioned, our results indicate that not only did irradiation increase those rates, but the impact was even more apparent when FCCP was present and sperm were light-stimulated for 1 min. Regarding the mechanism/s through which irradiation with red-light increases O_2_ consumption rates, we could raise two hypothesizes. The first one would be related to a direct effect of light on some point/s of the Krebs cycle complex. In fact, proteins such as pyruvate dehydrogenase have been reported to be sensitive to light [55]. Following this, one could suggest that irradiation could act on these light-sensitive structures of the Krebs cycle, modifying their activity, which would be reliant upon the light-stimulation pattern. On the other hand, our results also suggest that FCCP has an additive effect on the acceleration of the Krebs cycle, which could be related to the ability of FCCP to deregulate the electron chain, which would, in turn, lead to an uncontrolled acceleration of this system. Moreover, the absence of significant additive effects of FCCP on the O_2_ consumption rates of sperm irradiated for 5 or 10 min could be explained by the fact that these sperm cells would have already reached the maximal level, so that FCCP could not further increase these rates significantly. All these hypotheses support that further research on this subject is warranted.

The interpretation of results regarding the effects of light, oligomycin A and FCCP on the structure of motile sperm subpopulations is a very complex matter. In this respect, our results indicated that the presence of both oligomycin A and FCCP decreased the percentages of sperm with rapid motion characteristics, as the decrease in Subpopulations 2 and 3 showed. This was especially apparent when oligomycin A was present, which suggests that mitochondrial ATP production is very important to maintain fast sperm motility, despite the fact that the overall intracellular ATP levels were not significantly decreased. This effect of oligomycin A has already been described by our group under capacitating conditions [41], thus suggesting a relationship between sperm motility and the entire oxidative phosphorylation mechanism beyond the mere ATP synthesis. However, again, we can raise this hypothesis, but further investigations must be carried out to understand this phenomenon completely. Regarding the effects of irradiation, our data indicate that not only was irradiation unable to counteract the effects of both oligomycin A and FCCP, but it did not affect the percentages of slower sperm, as observed when sperm were irradiated for 5 min. These results differ from those obtained when sperm are irradiated for 10 min, allowed to rest for 10 min and irradiated again for 10 min, as published before [4]. An explanation for these differences could be that the precise effect of light would heavily rely upon the irradiation timing applied which, in turn, would be related with the specific energy load applied on cells. It is very likely that a specific and narrow energy load range could have specific effects on sperm mitochondrial function that would affect motility in one way, whereas other energy load ranges induced by different irradiation patterns could have other effects. In fact, the specific effects observed for each irradiation pattern (i.e., 1, 5 and 10 min) on the activity of cytochrome *c* oxidase would agree with that hypothesis. In effect, the applied irradiation regimes could modulate cytochrome *c* oxidase activity, which takes part in the mitochondrial electron chain; this could have concomitant, separate effects on other sperm function parameters that, such as sperm motility, are modulated by the electron chain and subsequent mitochondrial ATP synthesis. Indeed, taking into account that recent studies have demonstrated a strong relationship between mitochondrial ATP production and the regulation of sperm motility [42], one could reasonably suggest the specific energy load induced by irradiation is related to the specific rate of electron chain and motility patterns. However, further in-depth studies will be needed to verify this hypothesis.

The changes observed for the intracellular calcium levels are also difficult to explain. While no treatment affected the percentage of cells with high intramitochondrial calcium levels, the intensity of calcium stain was only increased when sperm cells were irradiated for 5 min. Remarkably, that augment was abolished by both oligomycin A and FCCP. While we cannot ascertain a clear reason for this phenomenon, one could suggest that it could be related to the intrinsic activity of the electron chain. In this respect, it is worth noting that irradiating pig sperm for 5 min induced a strong increase in the activity of CCO, which was also abolished by both oligomycin A and FCCP. This increase was concomitant with that of mitochondrial calcium, which suggests a direct relationship between the electron chain and intramitochondrial calcium levels. In fact, such a relationship between mitochondrial calcium and CCO activity has already been reported [56], thus suggesting a putative explanation for this observation. On the other hand, the decrease in Fluo3-intensity observed in sperm exposed to red-light for 10 min in the presence of FCCP is even more difficult to explain. In this case, a possible explanation could be related with some additional alterations caused by the modification of the electron chain activity induced by FCCP. Nevertheless, since the basis of this specific alteration remains unknown, further investigations are required.

Another interesting finding is that the effects of red LED light depend on the time of exposure. Related with this, our data are in agreement with previous studies about the sperm response to different red LED light patterns in terms of thermal resistance and in vitro capacitation [4]. While the mechanisms underlying this different time-response remain to be elucidated, it is reasonable to assume that the energy supplied to the mitochondrial electron chain by red-light is proportional to the time of exposure and intensity. It is worth mentioning that each specific photon has different energy potential levels depending on the wavelength. Since energy goes from high levels at the ultraviolet range to low levels at the infrared one, photons in the red range utilized herein have relatively low energy levels (http://www.photon.t.u-tokyo.ac.jp/~maruyama/nanoheat/Energy.pdf). Because the intensity of the device was kept constant in all protocols and wavelength ranged from 620 to 630 nm, the different pattern effects observed herein are likely to be due to the different times of exposure, which would fuel separate energy levels to mitochondrial photosensitizers. The energy supplied to photosensitizers modulate their rate of e^-^/H^+^ flux [57]. As a consequence, it is reasonable to hypothesize that energy-linked, light-induced changes on the electron chain activity affect the overall mitochondria function, which leads to variations in sperm motility and intracellular ion fluxes [57]. The final consequence is that different exposure times lead to a distinct impact on sperm function, depending on the exact energy level and the rate at which this energy is fueled into mitochondria. A corollary of this hypothesis is that any condition affecting the number of photons that reach cytochromes exerts a different impact on the sperm cell. In this context, it is worth mentioning that the sperm response to red-light has been suggested to be reliant upon other factors, such as the recipient in which sperm are contained [6]. However, explaining these effects should also take into consideration other potential sources of variation. The utilization of the same device and the same light regime for sperm stored in similar recipients regarding volume and color has been reported to render distinct effects on the overall sperm function [6,7]. In effect, differences in the storage time of sperm prior to light-stimulation between Luther et al. [6] and Pezo et al. [7] could explain these apparently inconsistent results. Whereas Luther et al. [6] stored sperm for 14 h prior to irradiation, that time lasted about 1 h in Pezo et al. [7]. This is important, since the functional status of pig sperm is different under these two conditions. In support of this hypothesis, cryotolerance of pig sperm when previously stored at 15–17 °C for 24 h is higher than when cryopreserved immediately after collection [58,59]. These differences have been linked with proteins related to the cell resilience to withstand environmental changes, such as heat shock protein 70 (HSPA1A), which shows much higher serine-phosphorylation levels after 24 h of storage at 15–17 °C [58]. Therefore, the functional sperm status is likely to lead to different sensitiveness to light-provided energy.

In spite of the aforementioned, there could be other factors that modulate the effects of red-light. This includes the presence of light-sensitive receptors, such as opsins [20] and transient receptor potential proteins (TRP; see [60,61,62]), in mammalian spermatozoa. While the exact role of these receptors in sperm is not completely known, the regulation of thermotaxis has been suggested to be one of their most probable functions [17,20]. Thermotaxis could be, in fact, an important modulator of light-stimulation, since mammalian sperm are sensitive to environmental temperature changes as low as 0.0006 °C [63]. Given that these small changes cannot be controlled by devices such as the one used to irradiate sperm in this study, it is reasonable to suggest that red-light stimulation could also act through inducing temperature changes higher than 0.0006 °C.

## 5. Conclusions

In conclusion, our results indicate that the effects induced by red LED light stimulation on mammalian sperm are related to mitochondrial photosensitizers, such as CCO, modifying mitochondrial electron chain activity. In addition, this study has also shown that the impact of red-light depends on the precise, previous sperm functional status and on the time of exposure. These findings do not exclude, however, that this mitochondrial mechanism acts together with thermotaxis via other receptors.

## Figures and Tables

**Table 1:** Effects of light-stimulation on kinematic sperm parameters in the presence or absence of either oligomycin A or FCCP.

	Non-Irradiated Samples			Irradiation for 1 min			Irradiation for 5 min			Irradiation for 10 min		
	Control	Oligomycin A	FCCP	Control	Oligomycin A	FCCP	Control	Oligomycin A	FCCP	Control	Oligomycin A	FCCP
VCL (µm/s)	70.6 ± 6.5 ^1a^	48.1 ± 3.8 ^1b^	54.0 ± 4.3 ^1b^	52.0 ± 4.0 ^2a^	50.7 ± 4.3 ^1a^	49.2 ± 4.6 ^1a^	65.6 ± 5.2 ^1a^	45.8 ± 3.9 ^1b^	52.0 ± 4.4 ^1b^	63.1 ± 5.0 ^1a^	49.1 ± 4.6 ^1b^	53.3 ± 4.7 ^1ab^
VSL (µm/s)	28.9 ± 2.6 ^1a^	19.5 ± 1.7 ^1b^	20.3 ± 1.8 ^1b^	22.6 ± 2.0 ^2a^	21.4 ± 1.9 ^1a^	20.2 ± 1.9 ^1a^	26.6 ± 2.0 ^12a^	18.1 ± 1.4 ^1b^	18.4 ± 1.5 ^1b^	26.6 ± 1.9 ^1 2a^	17.9 ± 1.3 ^1b^	17.8 ± 1.4 ^1b^
VAP (µm/s)	51.2 ± 5.5 ^1a^	29.7 ± 2.6 ^1b^	31.1 ± 2.9 ^1b^	36.3 ± 3.0 ^2a^	32.4 ± 3.0 ^1a^	30.8 ± 2.6 ^1a^	42.0 ± 4.4 ^12a^	27.0 ± 2.6 ^1b^	29.7 ± 3.1 ^1b^	42.9 ± 4.8 ^12a^	27.5 ± 2.6 ^1b^	29.8 ± 3.0 ^1b^
LIN (%)	42.0 ± 3.0 ^1a^	42.6 ± 3.1 ^1a^	39.6 ± 2.7 ^1a^	45.5 ± 3.5 ^1a^	44.2 ± 3.0 ^1a^	43.0 ± 2.6 ^1a^	42.6 ± 3.2 ^1a^	41.5 ± 3.0 ^1a^	37.4 ± 2.3 ^12b^	44.1 ± 3.9 ^1a^	38.5 ± 3.0 ^1a^	34.4 ± 2.1 ^2b^
STR (%)	58.4 ± 3.4 ^1a^	62.7 ± 4.3 ^1a^	63.3 ± 3.0 ^1a^	63.3 ± 3.2 ^1a^	64.0 ± 3.9 ^1a^	63.6 ± 3.6 ^1a^	61.3 ± 3.5 ^1a^	68.0 ± 4.0 ^1a^	63.9 ± 2.9 ^1a^	64.0 ± 3.5 ^1a^	67.1 ± 3.9 ^1a^	61.7 ± 3.1 ^1a^
WOB (%)	72.9 ± 4.9 ^1a^	63.7 ± 4.1 ^1ab^	59.6 ± 4.4 ^1b^	70.5 ± 4.9 ^1a^	67.4 ± 4.3 ^1a^	63.4 ± 3.8 ^1a^	66.1 ± 5.2 ^1a^	60.9 ± 4.1 ^1a^	58.9 ± 4.0 ^1a^	70.6 ± 4.8 ^1a^	58.0 ± 4.2 ^1b^	57.1 ± 4.9 ^1b^
ALH (µm)	2.78 ± 0.11 ^1a^	2.22 ± 0.09 ^1b^	2.49 ± 0.12 ^1b^	2.15 ± 0.08 ^2a^	2.26 ± 0.10 ^1a^	2.21 ± 0.10 ^2a^	2.44 ± 0.12 ^3a^	2.14 ± 0.09 ^1b^	2.40 ± 0.11 ^1 2a^	2.53 ± 0.12 ^1 3a^	2.28 ± 0.12 ^1b^	2.47 ± 0.14 ^1ab^
BCF (Hz)	7.7 ± 0.8 ^1a^	6.7 ± 0.4 ^1a^	7.7 ± 0.7 ^1a^	7.3 ± 1.0 ^1a^	7.5 ± 0.8 ^1a^	7.5 ± 0.7 ^1a^	7.4 ± 0.6 ^1a^	7.2 ± 0.8 ^1a^	7.2 ± 0.9 ^1a^	8.5 ± 0.8 ^1a^	7.5 ± 0.4 ^1a^	7.7 ± 0.8 ^1a^
DNC (µm^2^/s)	198.3 ± 10.9 ^1a^	108.8 ± 5.0 ^1b^	136.5 ± 7.2 ^1c^	119.8 ± 5.5 ^2a^	124.6 ± 4.9 ^2a^	108.7 ± 4.6 ^2b^	160.1 ± 9.6 ^3a^	108.1 ± 4.4 ^1b^	134.8 ± 5.8 ^1c^	169.6 ± 8.6 ^3a^	119.1 ± 4.8 ^12b^	141.7 ± 5.0 ^1c^
absMAD (°)	95.8 ± 6.1 ^1a^	106.9 ± 6.9 ^1a^	107.0 ± 7.0 ^1a^	97.6 ± 5.9 ^1a^	105.2 ± 6.9 ^1a^	107.2 ± 6.9 ^1a^	81.1 ± 5.4 ^2a^	115.3 ± 7.9 ^1b^	114.7 ± 7.6 ^1b^	99.7 ± 6.3 ^1a^	113.7 ± 7.3 ^1b^	118.2 ± 8.6 ^1b^
algMAD (°)	0.03 ± 0.01 ^1a^	−1.83 ± 0.13 ^1b^	−0.35 ± 0.10 ^1c^	0.20 ± 0.16 ^1 2a^	0.30 ± 0.18 ^2a^	0.20 ± 0.17 ^2a^	0.41 ± 0.17 ^2a^	−0.04 ± 0.10 ^3b^	0.24 ± 0.15 ^2a^	0.27 ± 0.18 ^2a^	1.05 ± 024 ^4b^	−0.56 ± 0.23 ^1c^

The analysis was conducted on a total number of 20,966 motile spermatozoa. Different superscript numbers indicate significant (*p* < 0.05) differences between light-stimulation patterns within a given treatment (i.e., control, oligomycin A or FCCP). Different superscript letters indicate significant (*p* < 0.05) differences between treatments within a given irradiation pattern (non-irradiated, light-stimulation for 1 min, light-stimulation for 5 min or light-stimulation for 10 min). Results are expressed as mean ± S.E.M. for seven experiments. Abbreviations: VCL, curvilinear velocity; VSL, straight line velocity; VAP, average pathway velocity; LIN, linearity coefficient; STR, straightness coefficient; WOB, wobble (oscillation) coefficient; ALH, amplitude of lateral head displacement; BCF, beat cross frequency; DNC, dance (VCL × ALH); absMAD, absolute mean angular displacement; algMAD, algebraic mean angular displacement.

## Supplementary Materials

The following are available online at https://www.mdpi.com/2073-4409/9/12/2546/s1. Supplementary Figure S1: Representative plots corresponding to the determination of O_2_ consumption rates in pig sperm incubated in the presence/absence of either oligomycin A or FCCP. A: Control. B: Sperm incubated with 5 µM oligomycin A. C: Sperm incubated with 5 µM FCCP. Supplementary Figure S2: Effects of light-stimulation on percentages of spermatozoa with an altered acrosome (PNA-FITC^-^). Sperm were not irradiated (non-irradiated), or irradiated for 1, 5 and 10 min. Control: sperm not incubated with either oligomycin A or FCCP. Oligomycin A: sperm incubated with 5 µM oligomycin A. FCCP: sperm incubated with 5 µM FCCP. No significant differences (*p* > 0.05) between treatments or light-stimulation patterns were observed. Results are expressed as mean ± SEM for seven separate experiments. Supplementary Figure S3: Representative histograms and dot-plots for the evaluation of JC1-staining (flow cytometry) in non-irradiated samples and samples irradiated for 1, 5 and 10 min. Results are shown separately for the control (A), and samples treated with oligomycin A (B) and FCCP (C). Supplementary Figure S4: Representative histograms and dot-plots for the evaluation of HE/YO-PRO-1-staining (flow cytometry) in non-irradiated samples and samples irradiated for 1, 5 and 10 min. Results are shown separately for the control (A), and samples treated with oligomycin A (B) and FCCP (C). Supplementary Figure S5: Representative histograms and dot-plots for the evaluation of H_2_DFCDA/PI-staining (flow cytometry) in non-irradiated samples and samples irradiated for 1, 5 and 10 min. Results are shown separately for the control (A), and samples treated with oligomycin A (B) and FCCP (C). Supplementary Figure S6: Representative histograms and dot-plots for the evaluation of Fluo3/PI-staining (flow cytometry) in non-irradiated samples and samples irradiated for 1, 5 and 10 min. Results are shown separately for the control (A), and samples treated with oligomycin A (B) and FCCP (C). Supplementary Table S1: Descriptive parameters (mean ± SEM; range) of the three sperm subpopulations (SP1, SP2 and SP3) identified in this study. Supplementary File S1: Time-lapse imaging of non-irradiated pig sperm stained with JC1 (control samples). Red spots correspond to oxidized JC1 (JC1_agg_) and green spots correspond to non-oxidized JC1 (JC1_mon_). No irradiation protocol induced any change on the specific localization of JC1_agg_ in the mid-piece. Supplementary File S2: Time-lapse imaging of non-irradiated pig sperm stained with JC1 (samples treated with oligomycin A). Red spots correspond to oxidized JC1 (JC1_agg_) and green spots correspond to non-oxidized JC1 (JC1_mon_). No irradiation protocol induced any change on the specific localization of JC1_agg_ in the mid-piece. Supplementary File S3: Time-lapse imaging of non-irradiated pig sperm stained with JC1 (samples treated with FCCP). Red spots correspond to oxidized JC1 (JC1_agg_) and green spots correspond to non-oxidized JC1 (JC1_mon_). No irradiation protocol induced any change on the specific localization of JC1_agg_ in the mid-piece.
