# Supplementary material for: Red LED Light Acts on the Mitochondrial Electron Chain of Mammalian Sperm via Light-Time Exposure-Dependent Mechanisms
Source: Cells. 2020 Nov 26;9(12):2546. doi: 10.3390/cells9122546 (PMC7760120; doi:10.3390/cells9122546)
Supplement: Supplementary file 1 [file cells-09-02546-s001.zip › Suppl Table 1 Description SPs.docx]

**Supplementary Table 1.** Descriptive parameters (mean ± SEM; range) of the three sperm subpopulations (SP1, SP2 and SP3) identified in this study.

|  | **SP1** | | **SP2** | | **SP3** | |
| --- | --- | --- | --- | --- | --- | --- |
| N | 9259 | | 6559 | | 5148 | |
|  | Mean ± SEM | Range (min, max) | Mean ± SEM | Range (min, max) | Mean ± SEM | Range (min, max) |
| VCL (µm/s) | 43.10 ± 0.15 | (10.10, 82.60) | 109.75 ± 0.39 | (52.40, 246.20) | 82.11 ± 0.35 | (34.20, 198.50) |
| VSL (µm/s) | 17.07 ± 0.08 | (0.00, 36.10) | 24.66 ± 0.17 | (0.00, 96.00) | 48.29 ± 0.21 | (25.50, 132.40) |
| VAP (µm/s) | 26.12 ± 0.10 | (9.10, 69.90) | 69.48 ± 0.33 | (10.00, 187.40) | 63.56 ± 0.30 | (29.60, 182.90) |
| LIN (%) | 41.75 ± 0.18 | (0.00, 97.83) | 22.86 ± 0.13 | (0.00, 50.63) | 60.17 ± 0.18 | (35.68, 98.68) |
| STR (%) | 65.79 ± 0.20 | (0.00, 100.00) | 37.86 ± 0.24 | (0.00, 91.68) | 77.50 ± 0.16 | (38.17, 99.31) |
| WOB (%) | 62.21 ± 0.15 | (17.80, 100.00) | 62.96 ± 0.17 | (16.05, 95.80) | 77.67 ± 0.15 | (45.69, 100.00) |
| ALH (µm) | 2.12 ± 0.01 | (0.30, 4.10) | 4.49 ± 0.01 | (1.60, 10.00) | 3.00 ± 0.01 | (0.50, 6.70) |
| BCF (Hz) | 6.45 ± 0.03 | (0.00, 17.00) | 7.97 ± 0.04 | (0.00, 22.00) | 8.65 ± 0.04 | (0.00, 20.00) |
| DNC (µm^2^/s) | 99.84 ± 0.61 | (4.71, 299.60) | 523.68 ± 3.62 | (110.45, 2307.30) | 264.62 ± 2.18 | 15.99, 1280.59) |
| absMAD (°) | 108.65 ± 0.35 | (6.42, 257.49) | 105.73 ± 0.41 | (19.31, 231.50) | 90.00 ± 0.58 | (12.72, 270.68) |
| algMAD (°) | 0.11 ± 0.07 | (-39.38, 35.00) | -0.08 ± 0.10 | (-39.67, 37.96) | -0.06 ± 0.12 | (-39.92, 38.78) |

**Abbreviations**: VCL, curvilinear velocity; VSL, straight line velocity; VAP, average pathway velocity; LIN, linearity coefficient; STR, straightness coefficient; WOB, wobble (oscillation) coefficient; ALH, amplitude of lateral head displacement; BCF, beat cross frequency; DNC: dance (VCL×ALH); absMAD, absolute mean angular displacement; algMAD, algebraic mean angular displacement.
