# Supplementary figures and images for: Red LED Light Acts on the Mitochondrial Electron Chain of Mammalian Sperm via Light-Time Exposure-Dependent Mechanisms
Source: Cells. 2020 Nov 26;9(12):2546. doi: 10.3390/cells9122546 (PMC7760120; doi:10.3390/cells9122546)

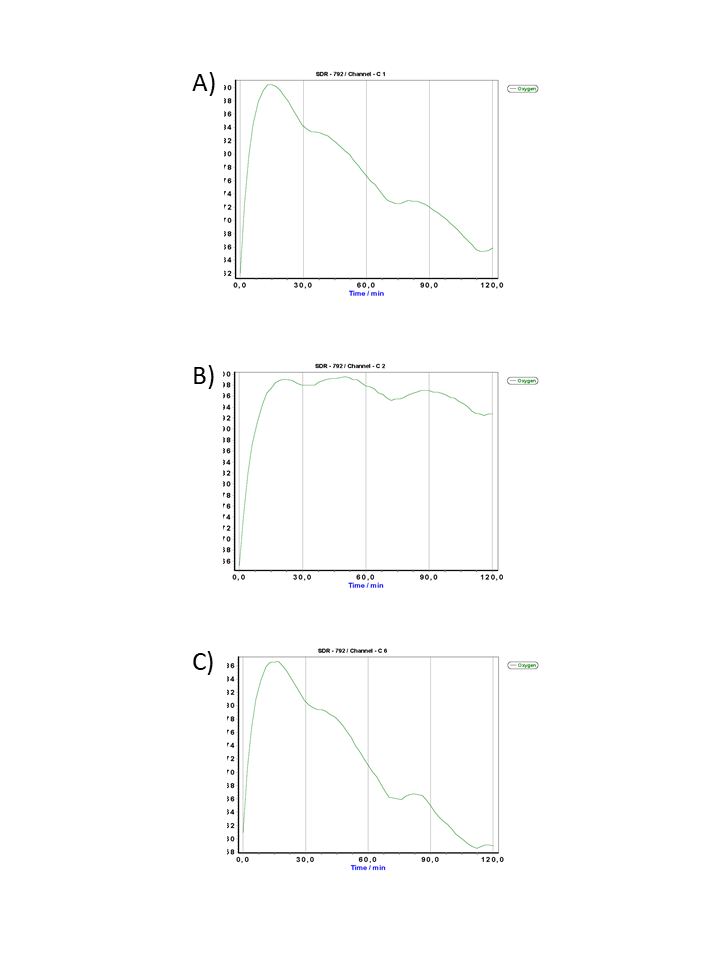

Supplement: Supplementary file 1 [file cells-09-02546-s001.zip › Suppl_Fig_1.tif]

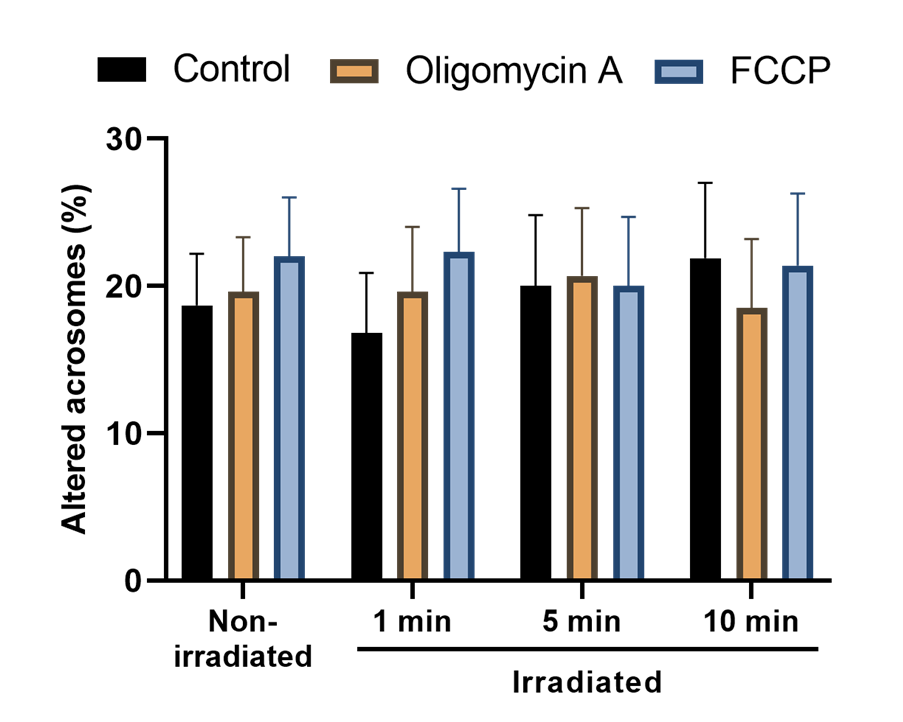

Supplement: Supplementary file 1 [file cells-09-02546-s001.zip › Suppl_Fig_2.tif]

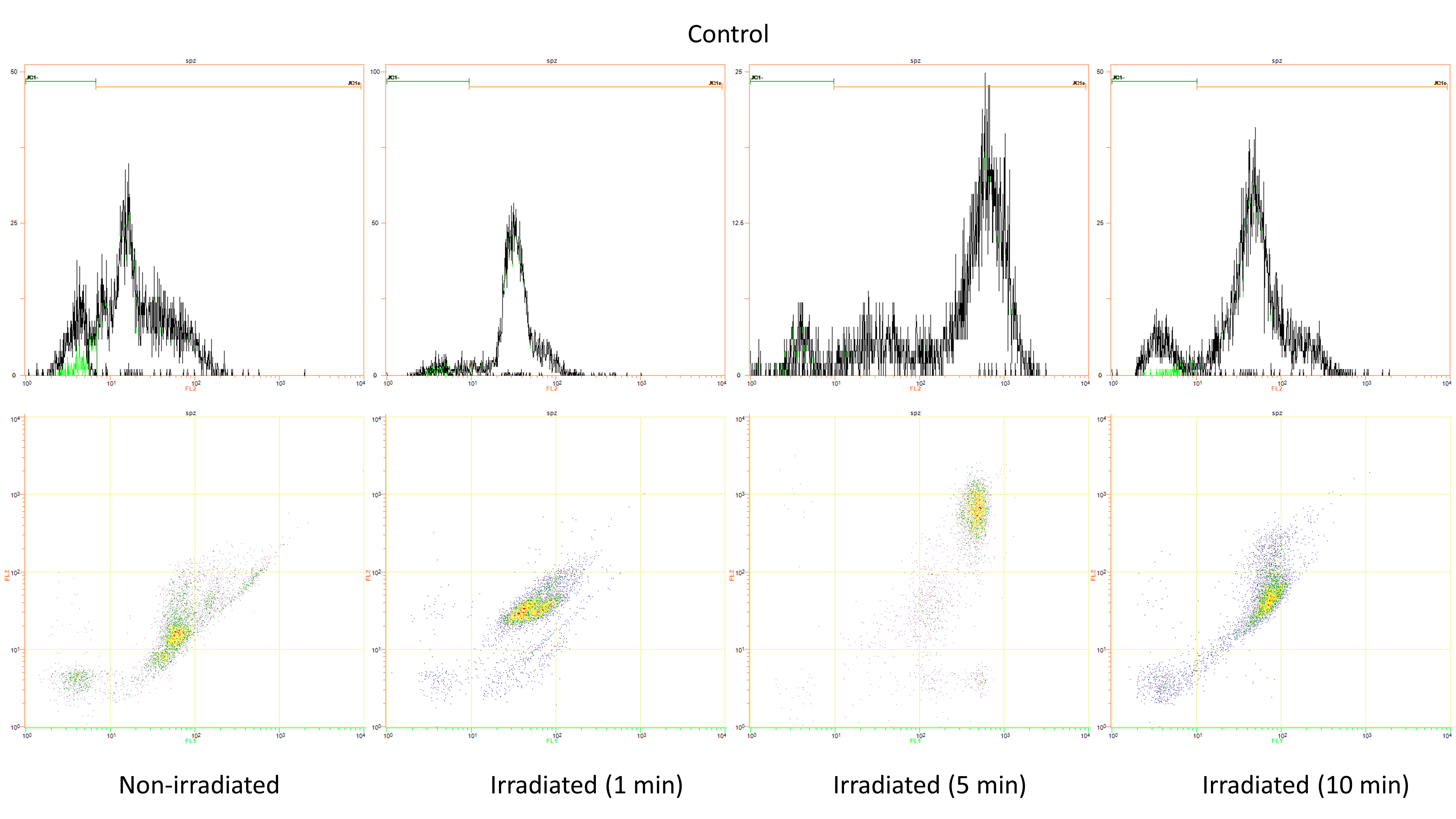

Supplement: Supplementary file 1 [file cells-09-02546-s001.zip › Suppl_Fig3_JC1_a.TIF]

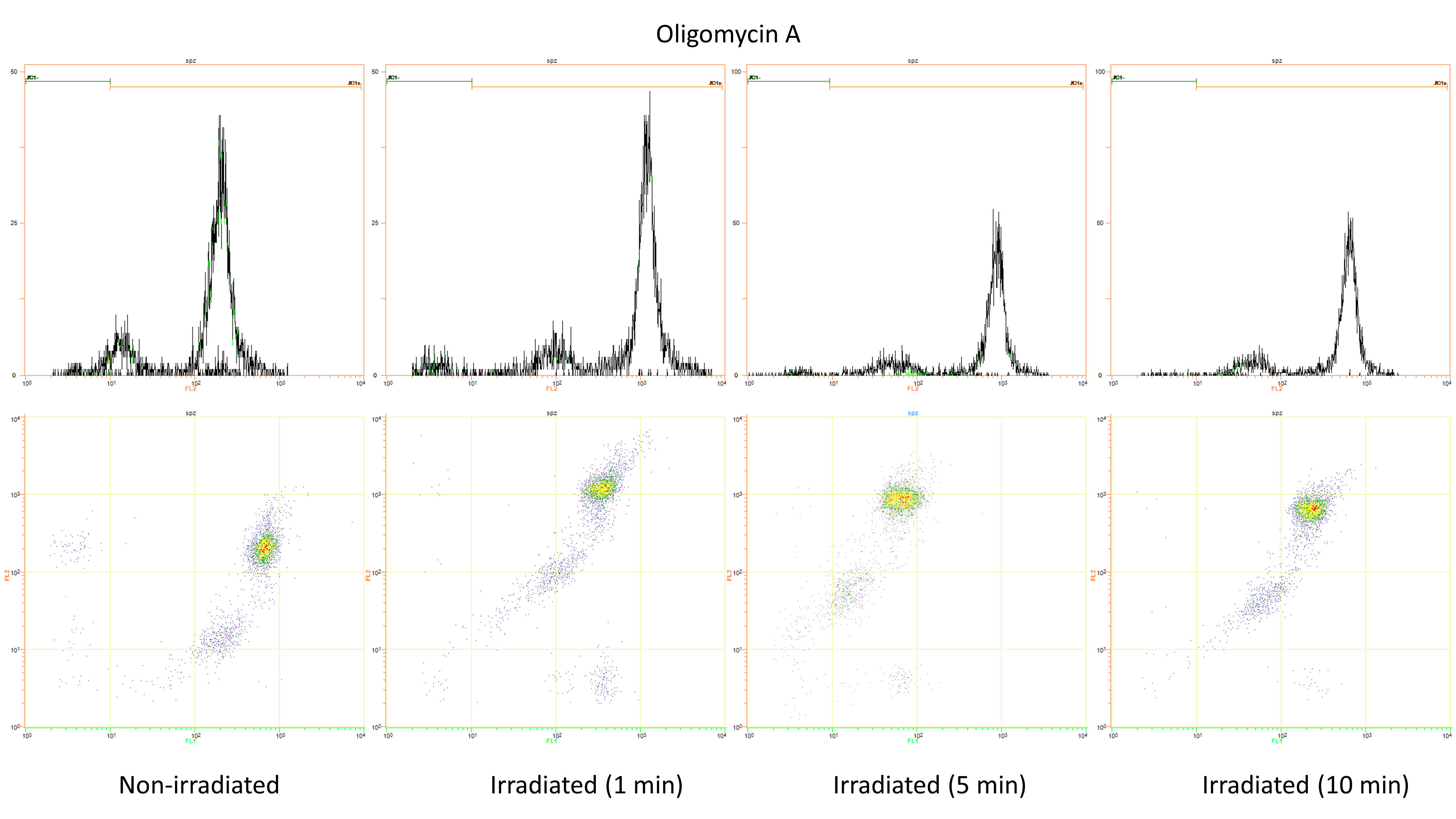

Supplement: Supplementary file 1 [file cells-09-02546-s001.zip › Suppl_Fig3_JC1_b.TIF]

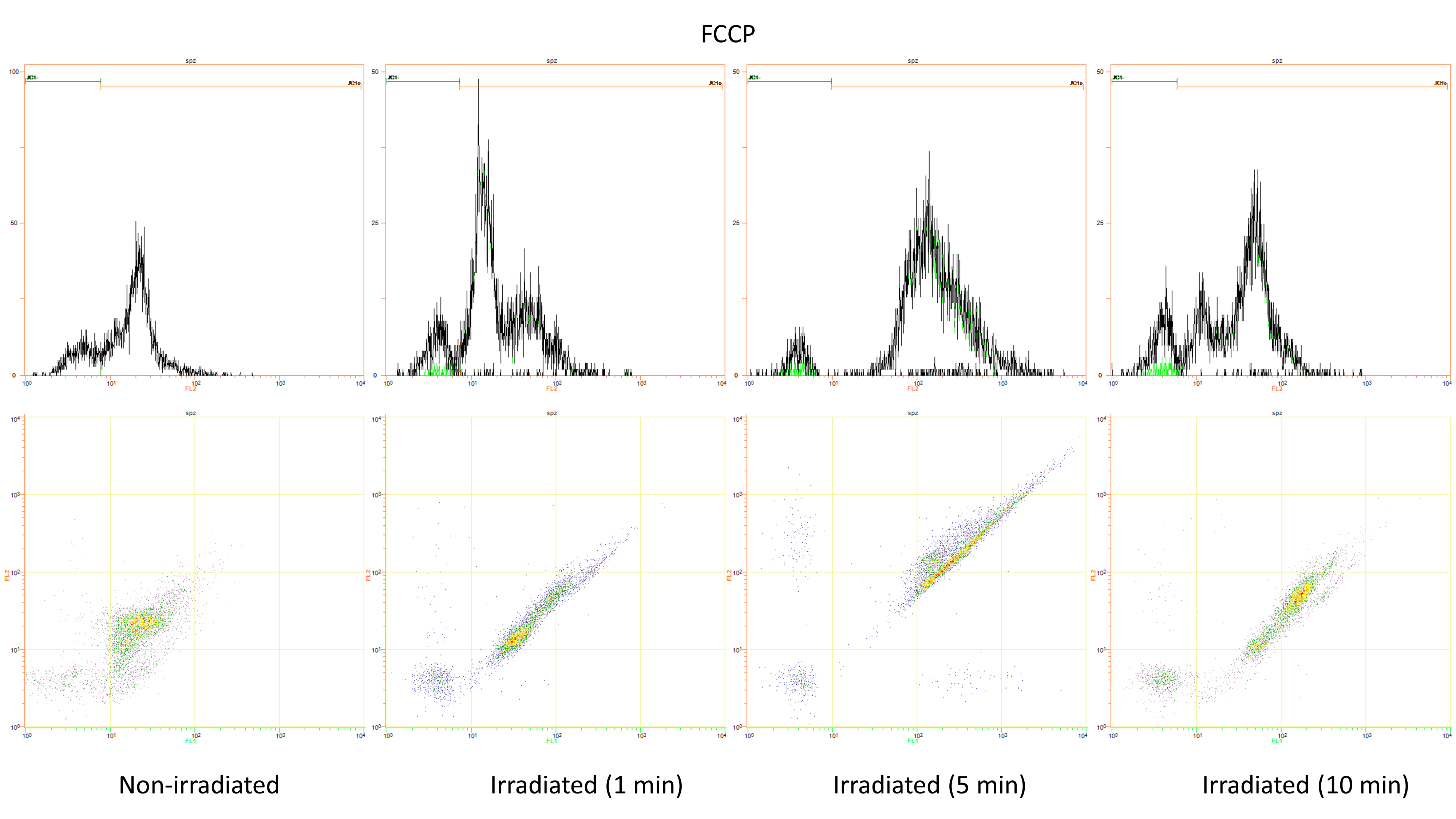

Supplement: Supplementary file 1 [file cells-09-02546-s001.zip › Suppl_Fig3_JC1_c.TIF]

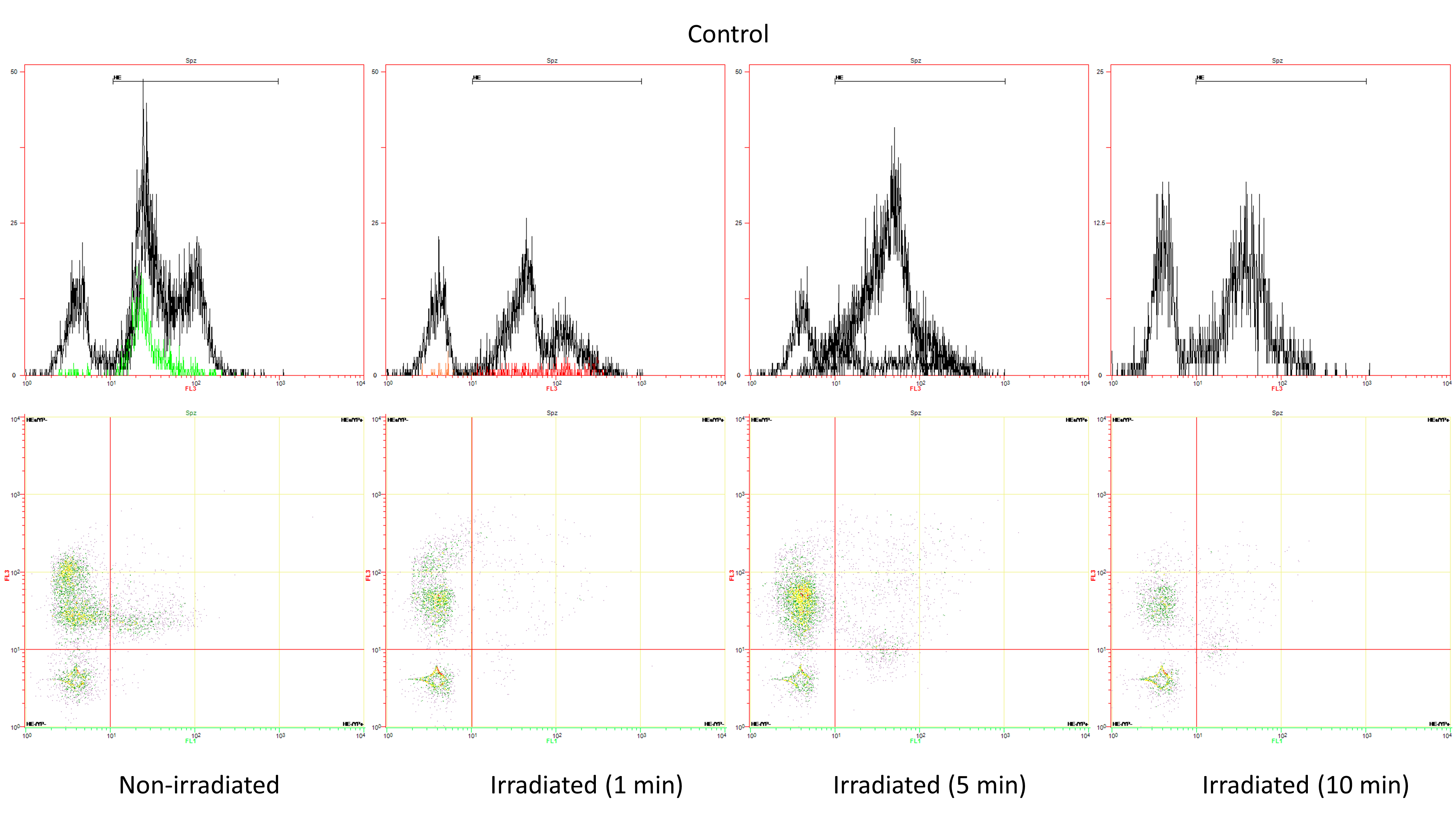

Supplement: Supplementary file 1 [file cells-09-02546-s001.zip › Suppl_Fig4_HE_a.TIF]

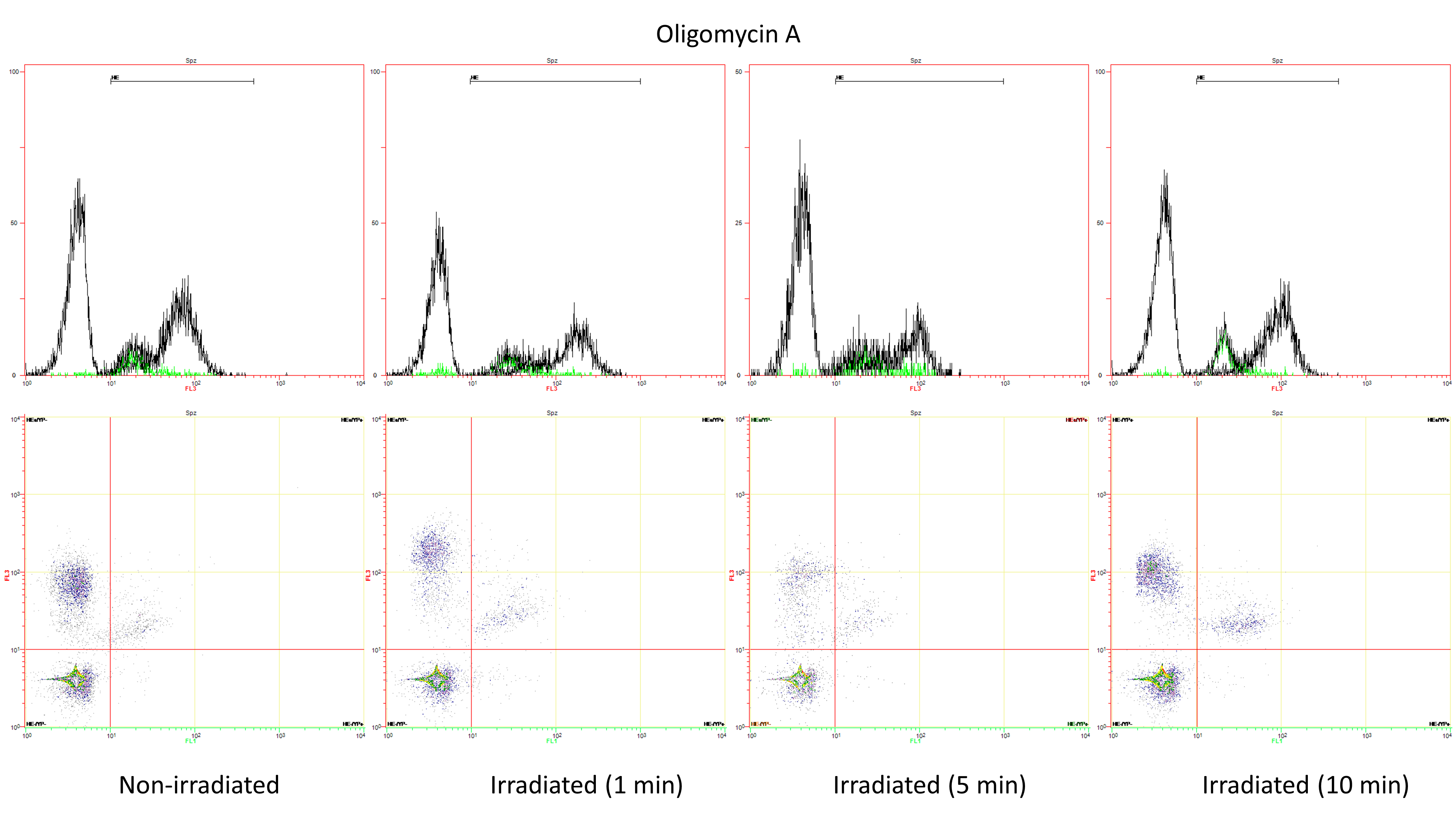

Supplement: Supplementary file 1 [file cells-09-02546-s001.zip › Suppl_Fig4_HE_b.TIF]

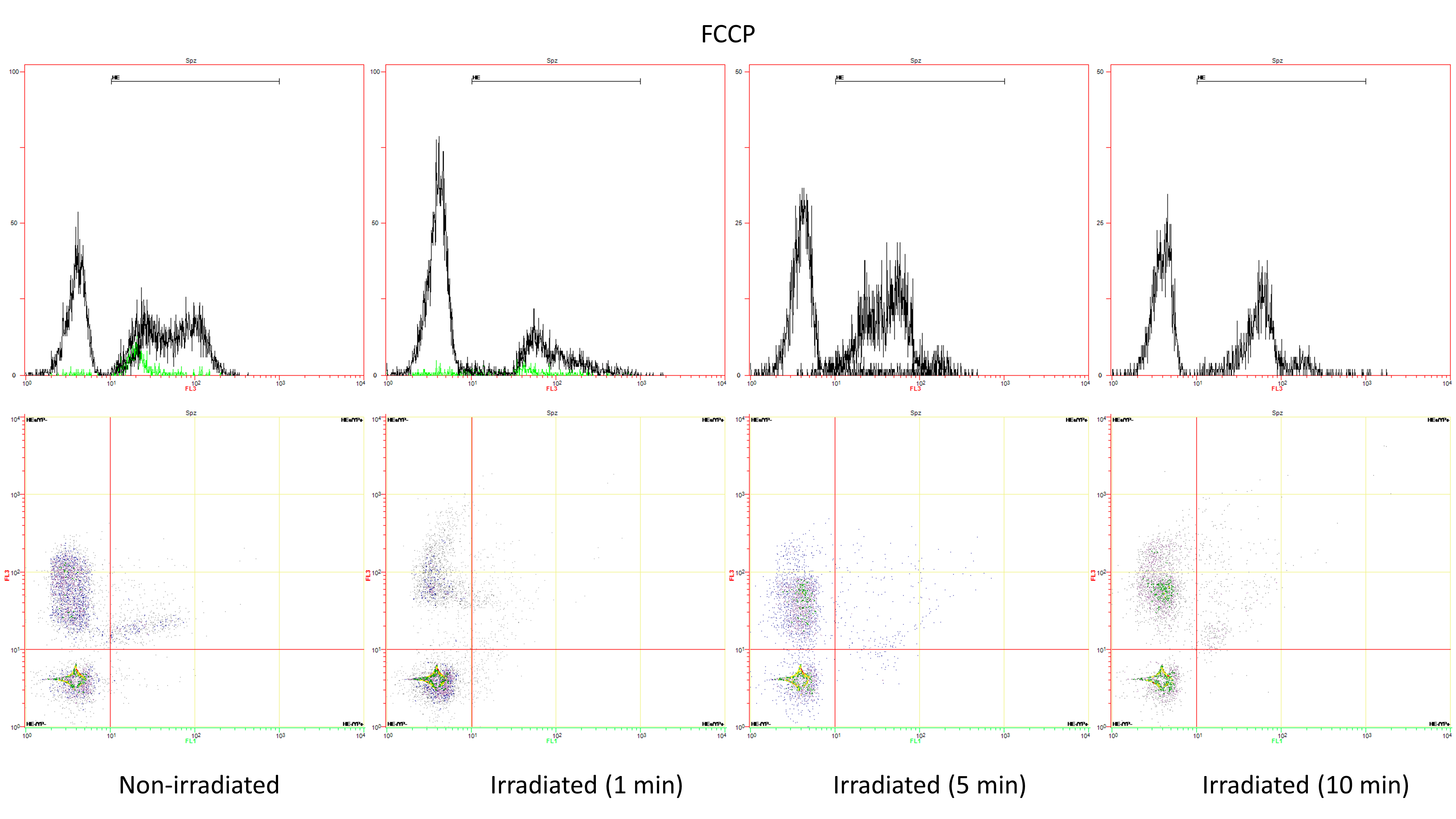

Supplement: Supplementary file 1 [file cells-09-02546-s001.zip › Suppl_Fig4_HE_c.TIF]

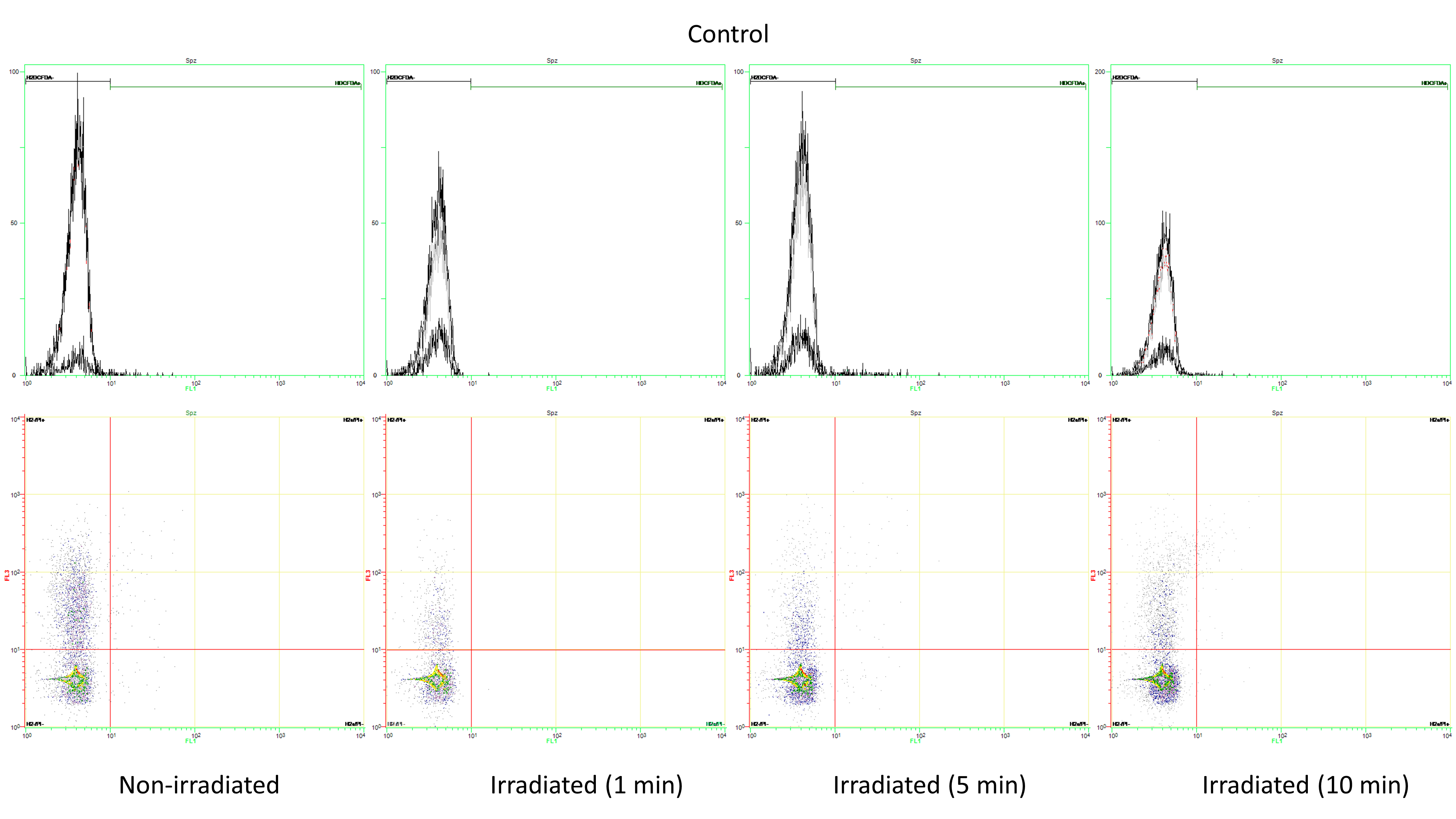

Supplement: Supplementary file 1 [file cells-09-02546-s001.zip › Suppl_Fig5_H2_a.TIF]

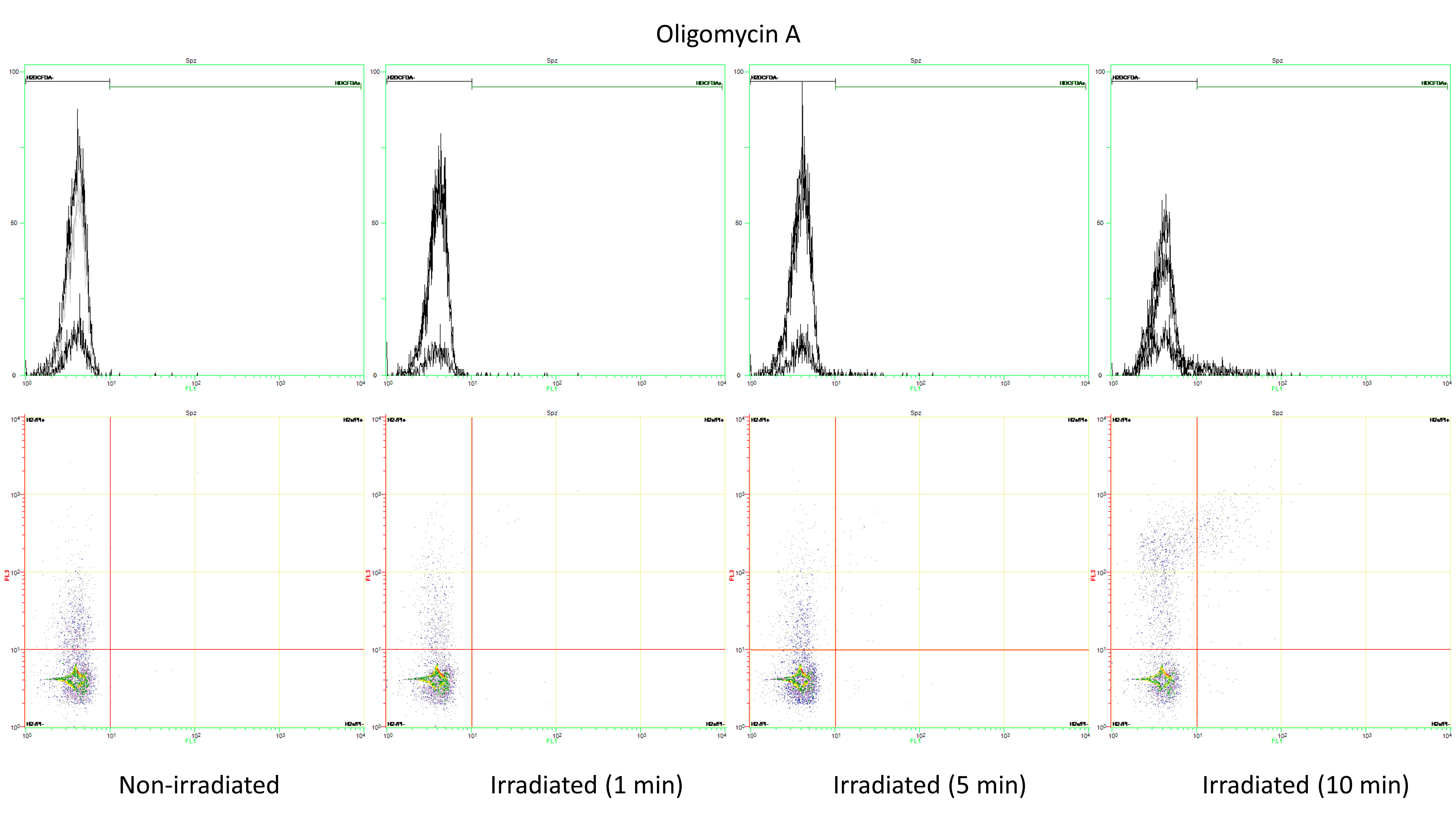

Supplement: Supplementary file 1 [file cells-09-02546-s001.zip › Suppl_Fig5_H2_b.TIF]

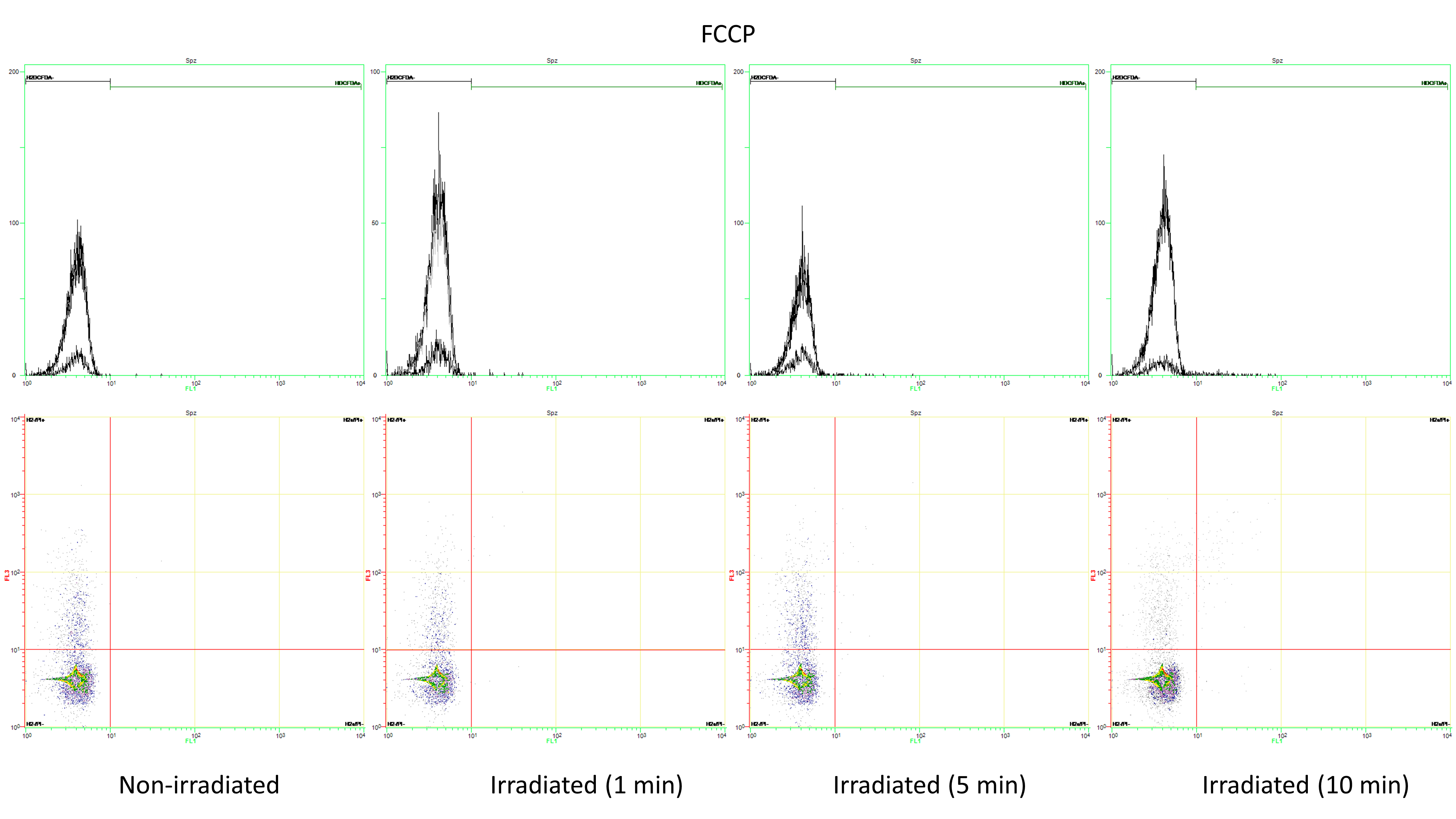

Supplement: Supplementary file 1 [file cells-09-02546-s001.zip › Suppl_Fig5_H2_c.TIF]

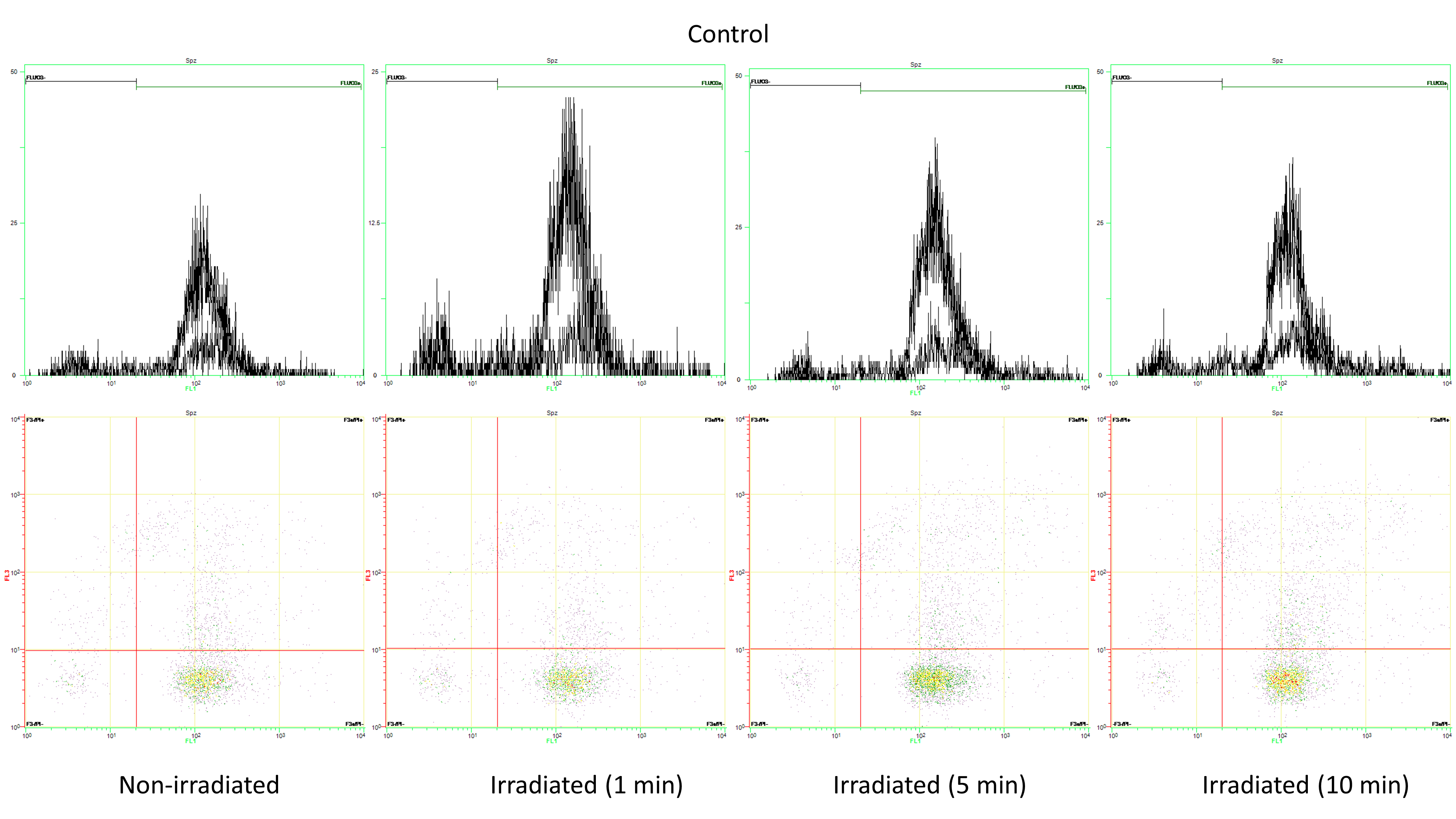

Supplement: Supplementary file 1 [file cells-09-02546-s001.zip › Suppl_Fig6_Fluo3_a.TIF]

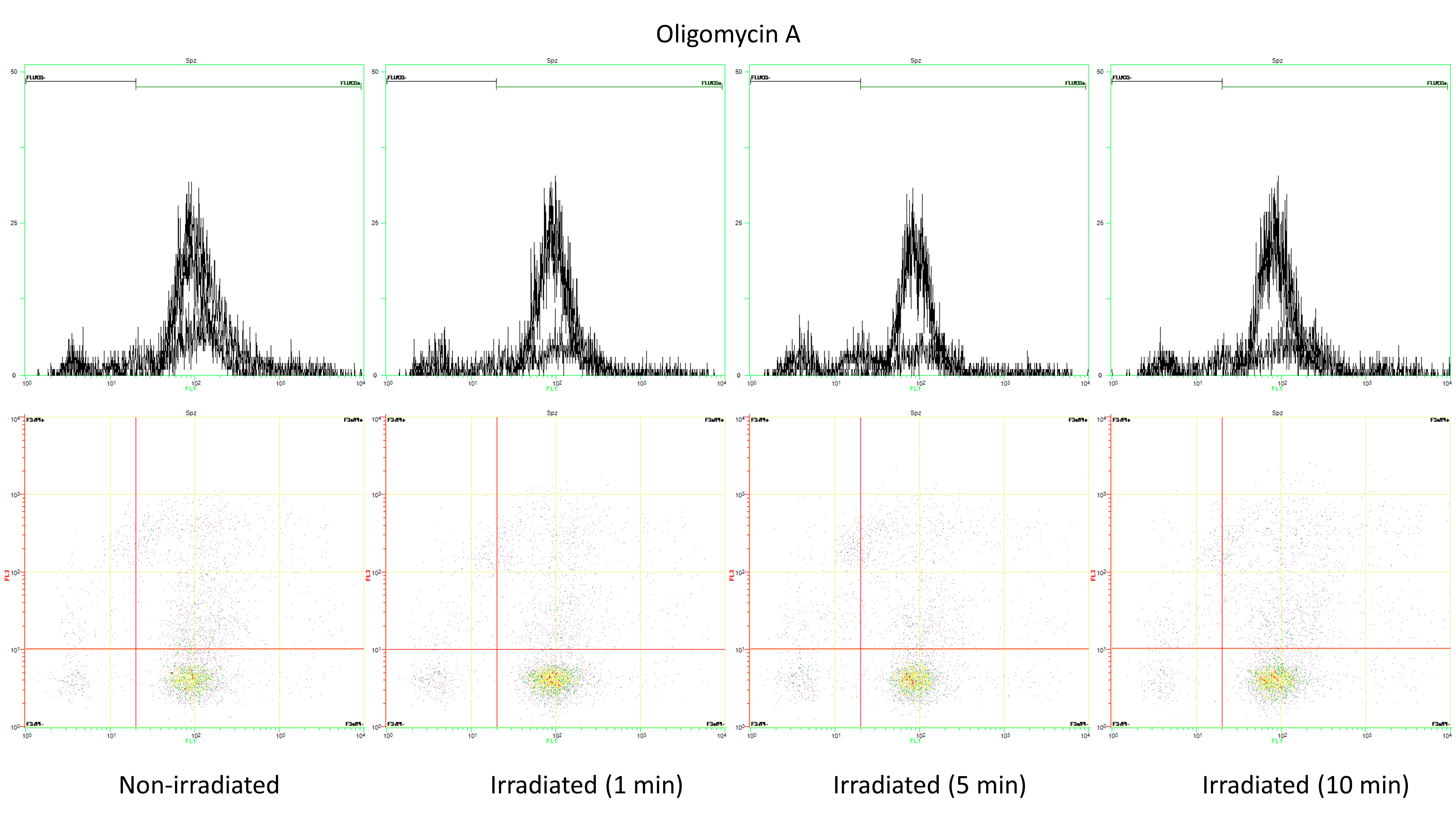

Supplement: Supplementary file 1 [file cells-09-02546-s001.zip › Suppl_Fig6_Fluo3_b.TIF]

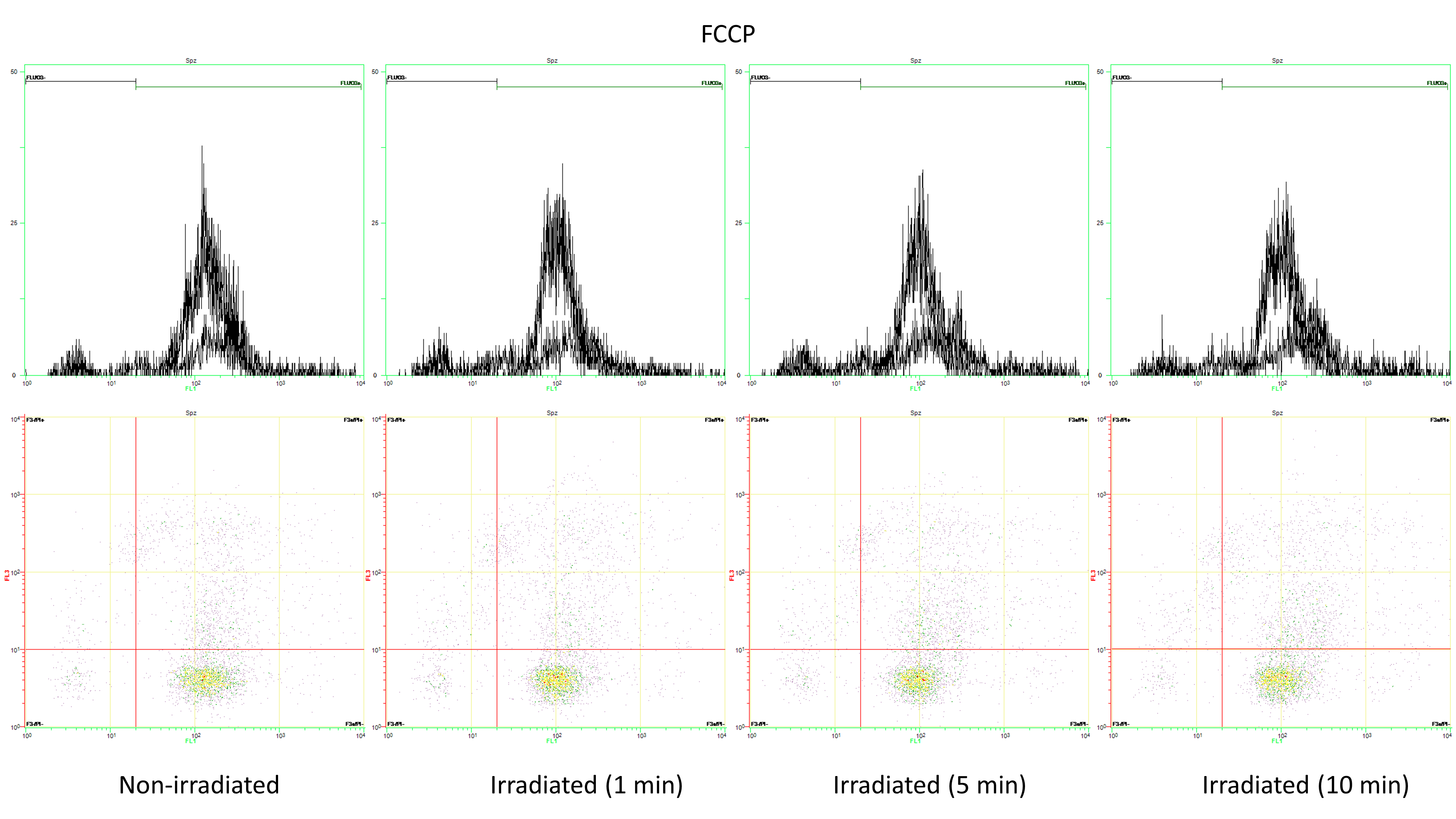

Supplement: Supplementary file 1 [file cells-09-02546-s001.zip › Suppl_Fig6_Fluo3_c.TIF]
